# Supplementary material for: HOXA9 inhibits HIF-1α-mediated glycolysis through interacting with CRIP2 to repress cutaneous squamous cell carcinoma development
Source: Nat Commun. 2018 Apr 16;9:1480. doi: 10.1038/s41467-018-03914-5 (PMC5902613; doi:10.1038/s41467-018-03914-5)
Supplement: Supplementary file 1 — Supplementary Information [file 41467_2018_3914_MOESM1_ESM.pdf]

Supplementary Information

HOXA9 Inhibits HIF-1α-Mediated Glycolysis through Interacting with CRIP2 to Repress Cutaneous Squamous Cell Carcinoma Development

Zhou, et.al.

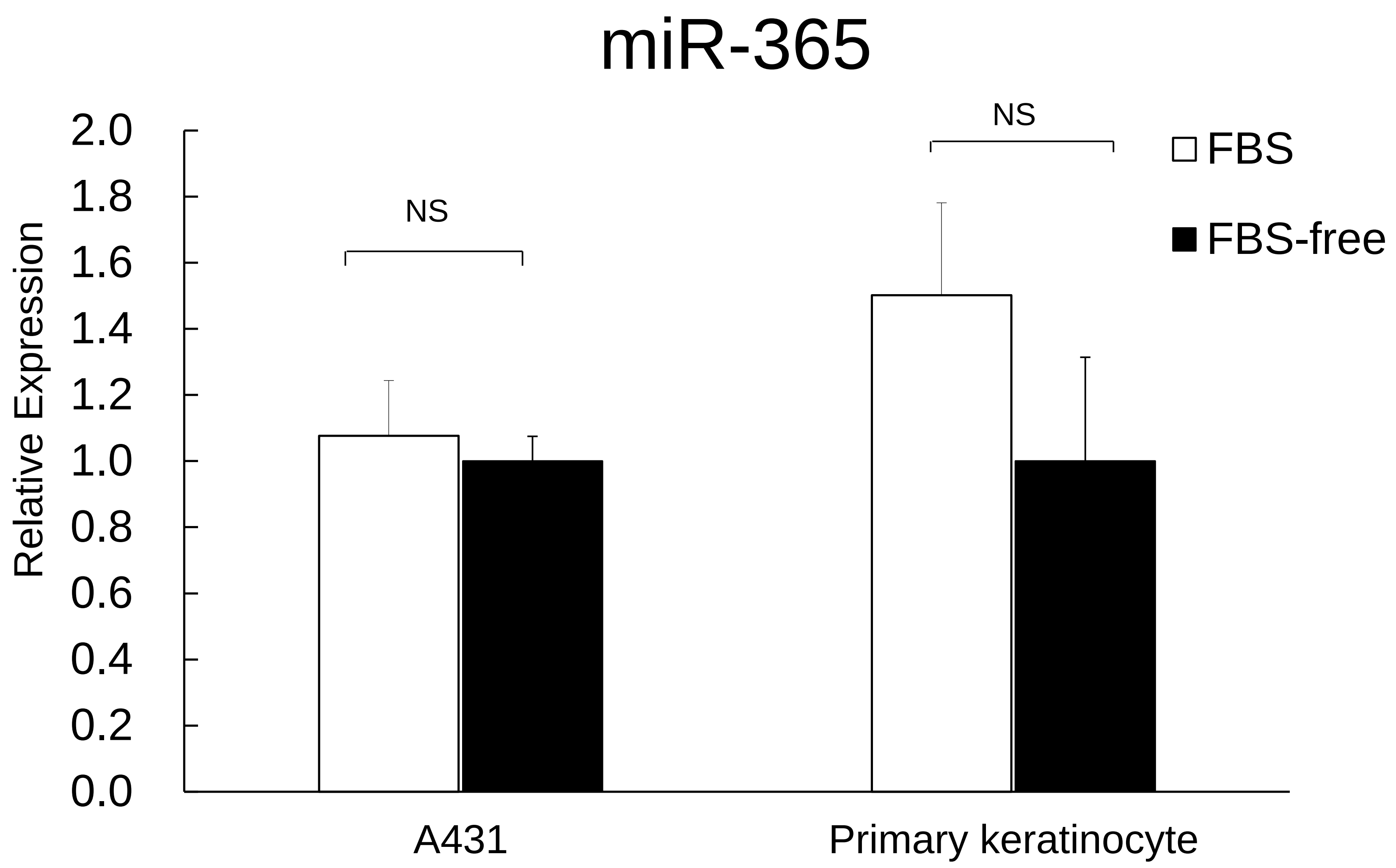

Supplementary Figure 1. Verification of miR-365 expression in response to the presence of FBS. No significant difference of the miR-365 expression levels between serum-free group and serum-containing group could be detected by qRT-PCR in both of the A431 cells and primary keratinocytes. NS, no significant difference. Each experiment was performed at least in triplicate and data are presented as mean ± s.d. One-Way ANOVA and Dunnett's multiple comparison test were used to analyze the data (*$P < 0.05$, **$P < 0.01$, ***$P < 0.001$).

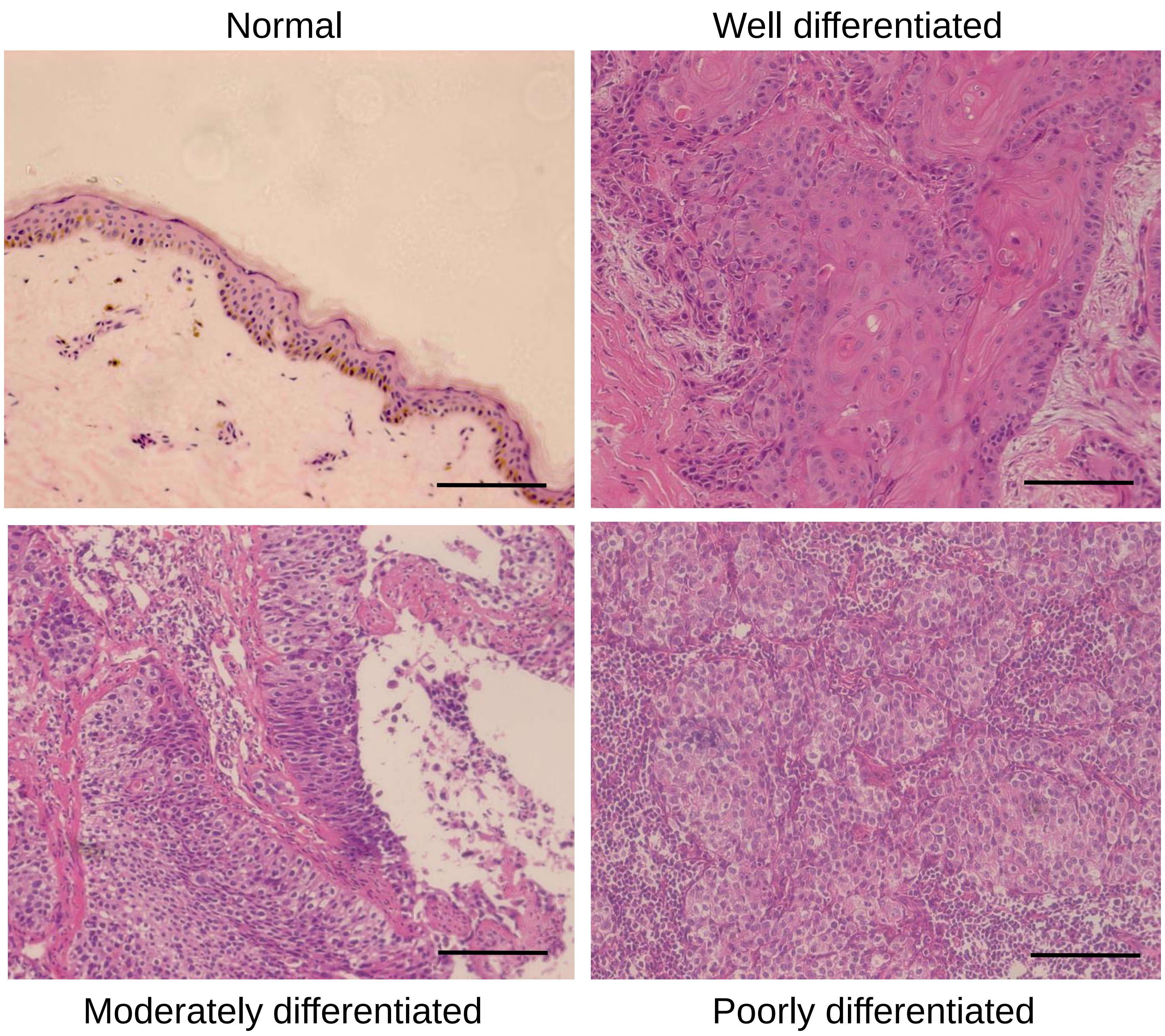

Supplementary Figure 2. Hematoxylin and eosin (H & E) staining on paraffin sections of cSCC tumors and normal skin specimens. Representative images indicating pathological cSCC tumor grades were shown: grade I (well differentiated), grade II (moderately differentiated) and grades III and IV (poorly differentiated). Scale bar, 100 μm. Each experiment was performed at least in triplicate.

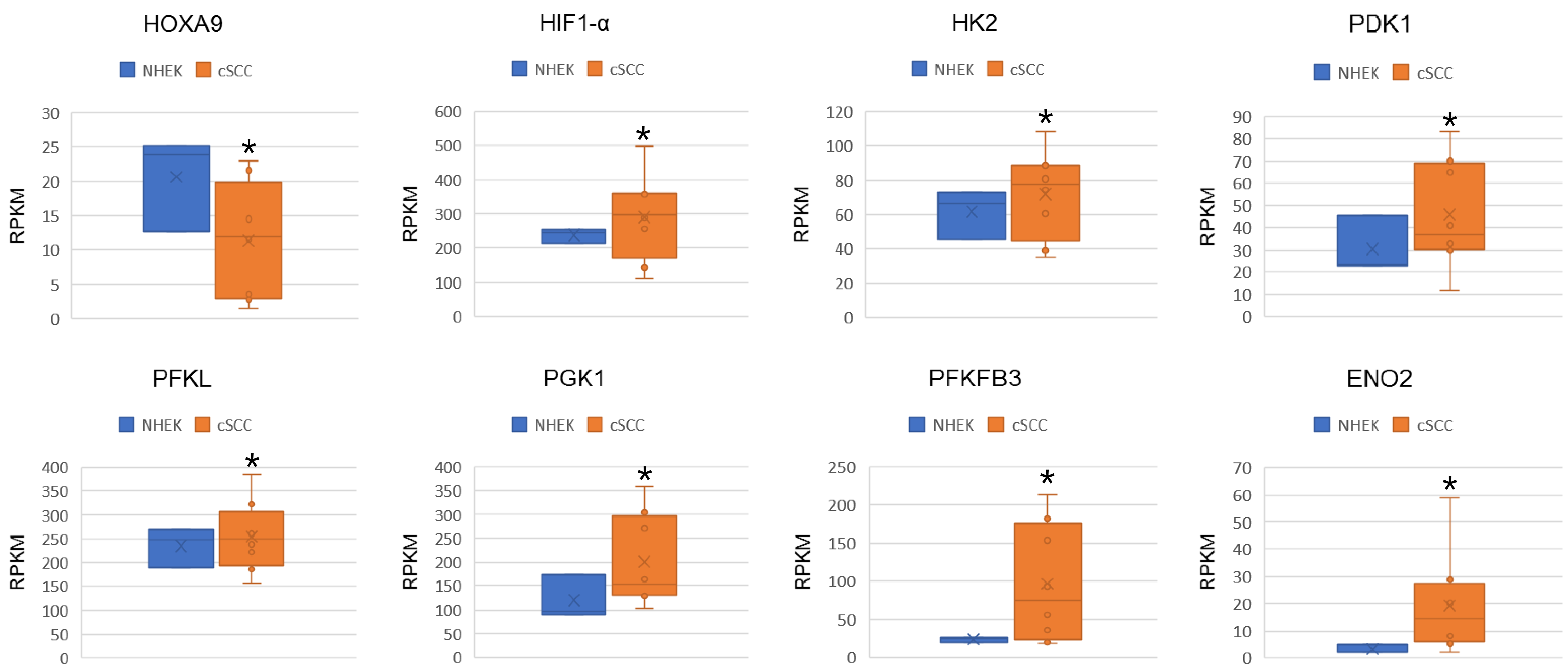

Supplementary Figure 3. Gene expression levels (Reads Per Kilobase per Million mapped reads, RPKM) of HOXA9, HIF-1α and its downstream glycolytic genes were compared between Normal Human Epidermal Keratinocytes (NHEK) (n=3) and cSCC tumors(n=8). Data are plotted as the means of 95% confidence interval ± s.d. One-Way ANOVA and Dunnett's multiple comparison test were used to analyze the data (*$P < 0.05$, **$P < 0.01$, ***$P < 0.001$).

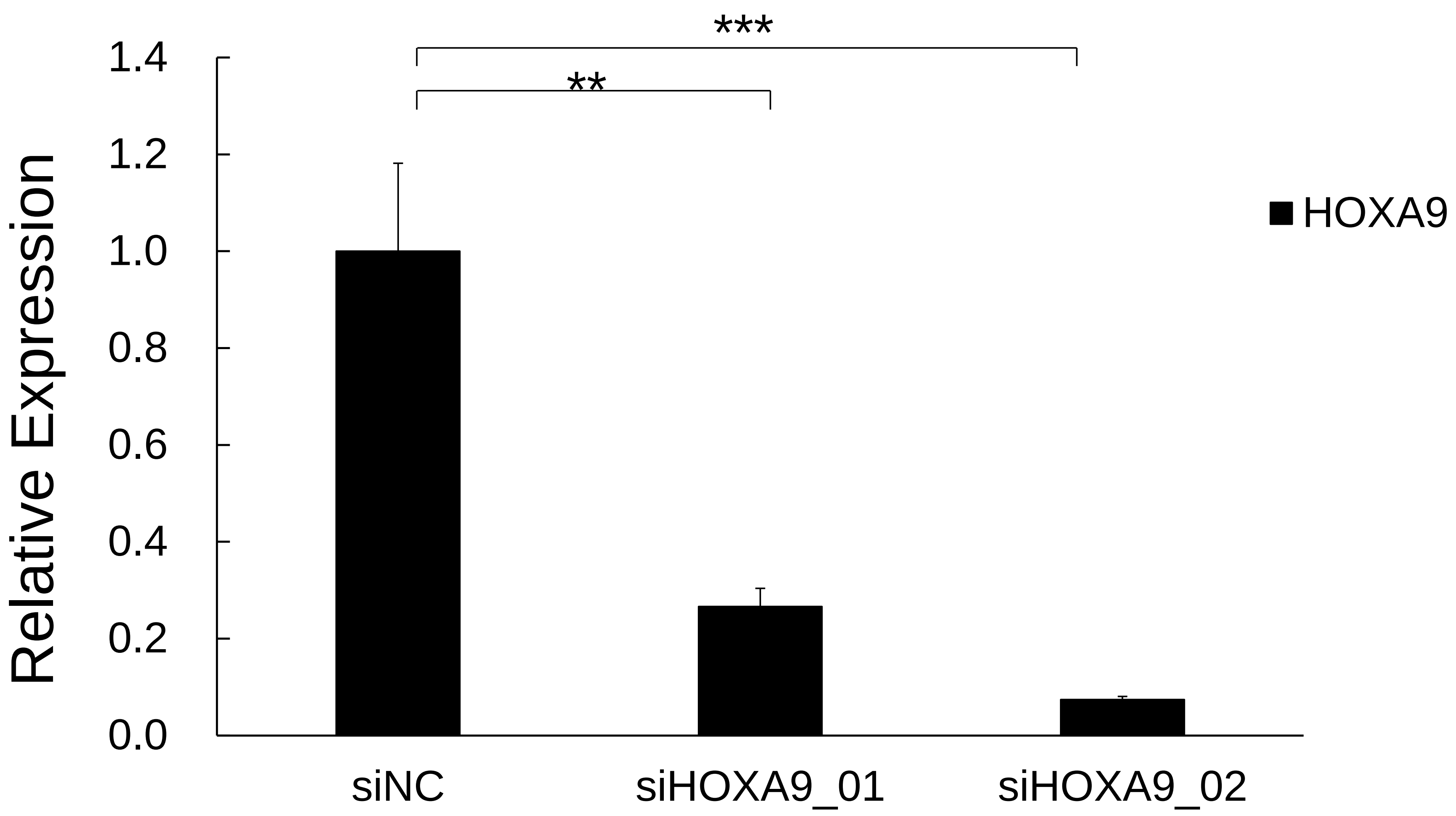

Supplementary Figure 4. qRT-PCR was performed and *HOXA9* mRNA expression levels were detected after depletion of HOXA9 by siRNAs in A431 cells. Significant loss of *HOXA9* mRNA expression can be observed. Each experiment was performed at least in triplicate and data are presented as mean ± s.d. One-Way ANOVA and Dunnett's multiple comparison test were used to analyze the data (*P < 0.05, **P < 0.01, ***P < 0.001).

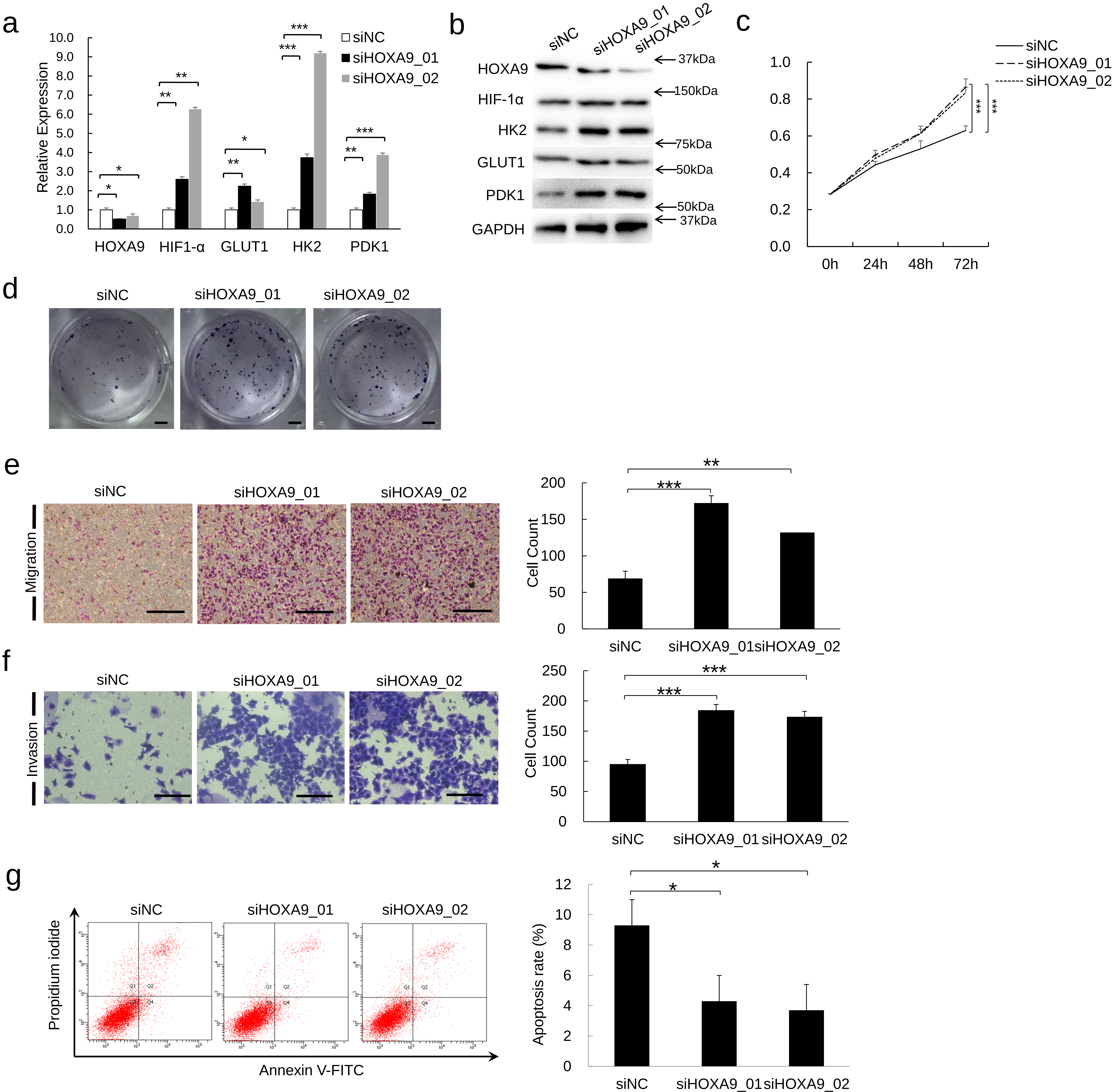

Supplementary Figure 5. Loss of HOXA9 promotes cell proliferation, migration, and invasiveness, but represses apoptosis in HSC-1 cells. (a,b) HOXA9 mRNA and protein expression was detected after depletion of HOXA9 by siRNAs in HSC-1 cells. Measurements of cell proliferation by CCK-8 assay (c), colony formation assay (d), transwell migration assay (e), Matrigel invasiveness measurement (f), and apoptosis assay by Annexin V/PI double staining (g) were performed in HSC-1 cells treated with siRNAs targeting HOXA9. In (d), scale bar : 1cm. In (e) and (f), scale bar: 100 µm. Each experiment was performed at least in triplicate and data are presented as mean ± s.d. One-Way ANOVA and Dunnett's multiple comparison test were used to analyze the data (**P* < 0.05, ***P* < 0.01, ****P* < 0.001).

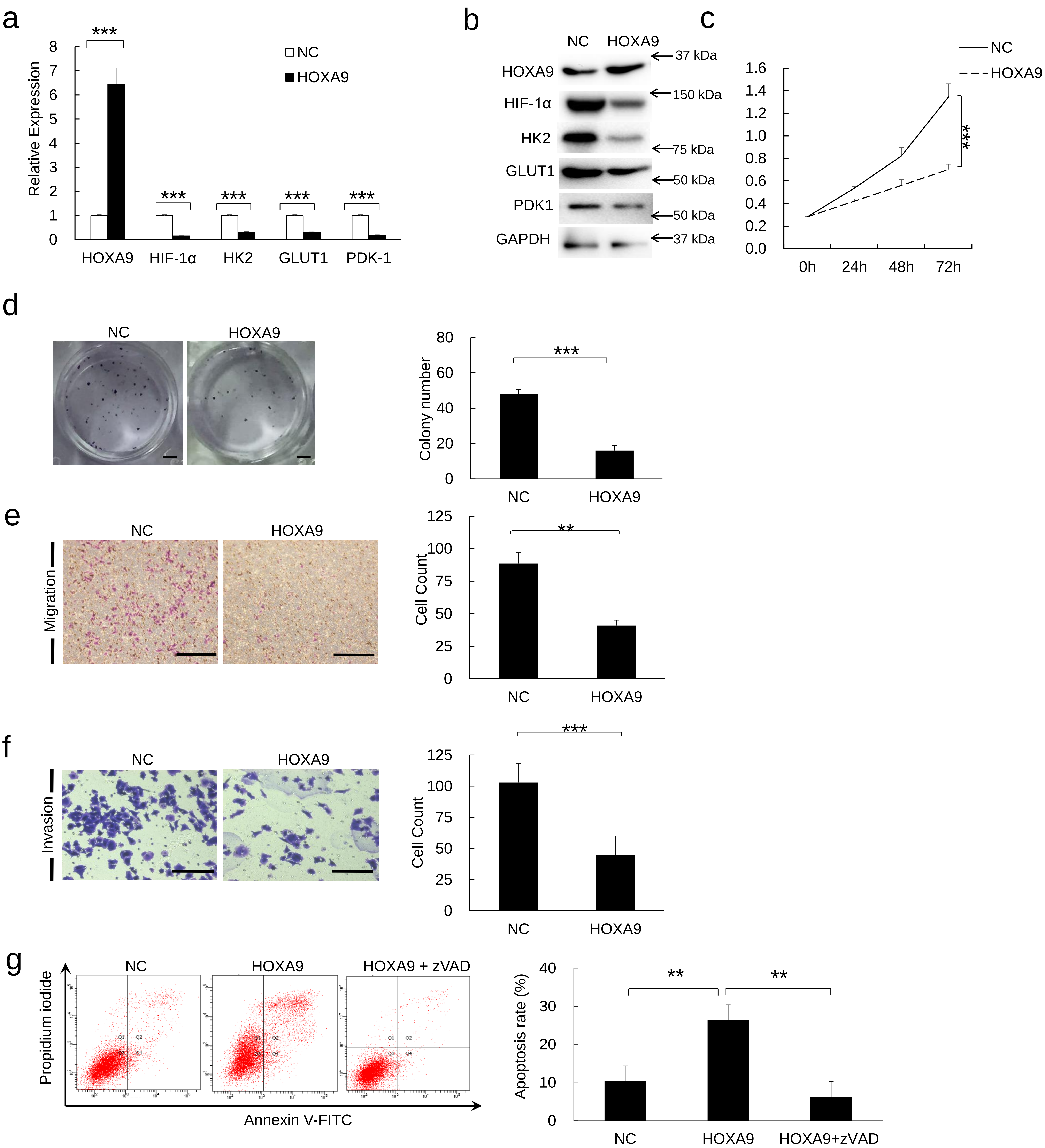

Supplementary Figure 6. HOXA9 represses cell proliferation, migration, and invasiveness, but promotes apoptosis in HSC-1 cells. (a,b) HOXA9 mRNA and protein expression was detected by qRT-PCR or western blot after overexpression of HOXA9 in HSC-1 cells. Measurements of cell proliferation by CCK-8 assay (c), colony formation assay (d), transwell migration assay (e), Matrigel invasiveness measurement (f), and apoptosis assay by Annexin V/PI double staining (g) were performed in HSC-1 cells overexpressing HOXA9. In (g), zVAD treatment was performed after HOXA9 overexpression to check the variation of apoptosis. In (d), scale bar : 1cm. In (e) and (f), scale bar: 100 μm. Each experiment was performed at least in triplicate and data are presented as mean ± s.d. One-Way ANOVA and Dunnett's multiple comparison test were used to analyze the data (**P* < 0.05, ***P* < 0.01, ****P* < 0.001).

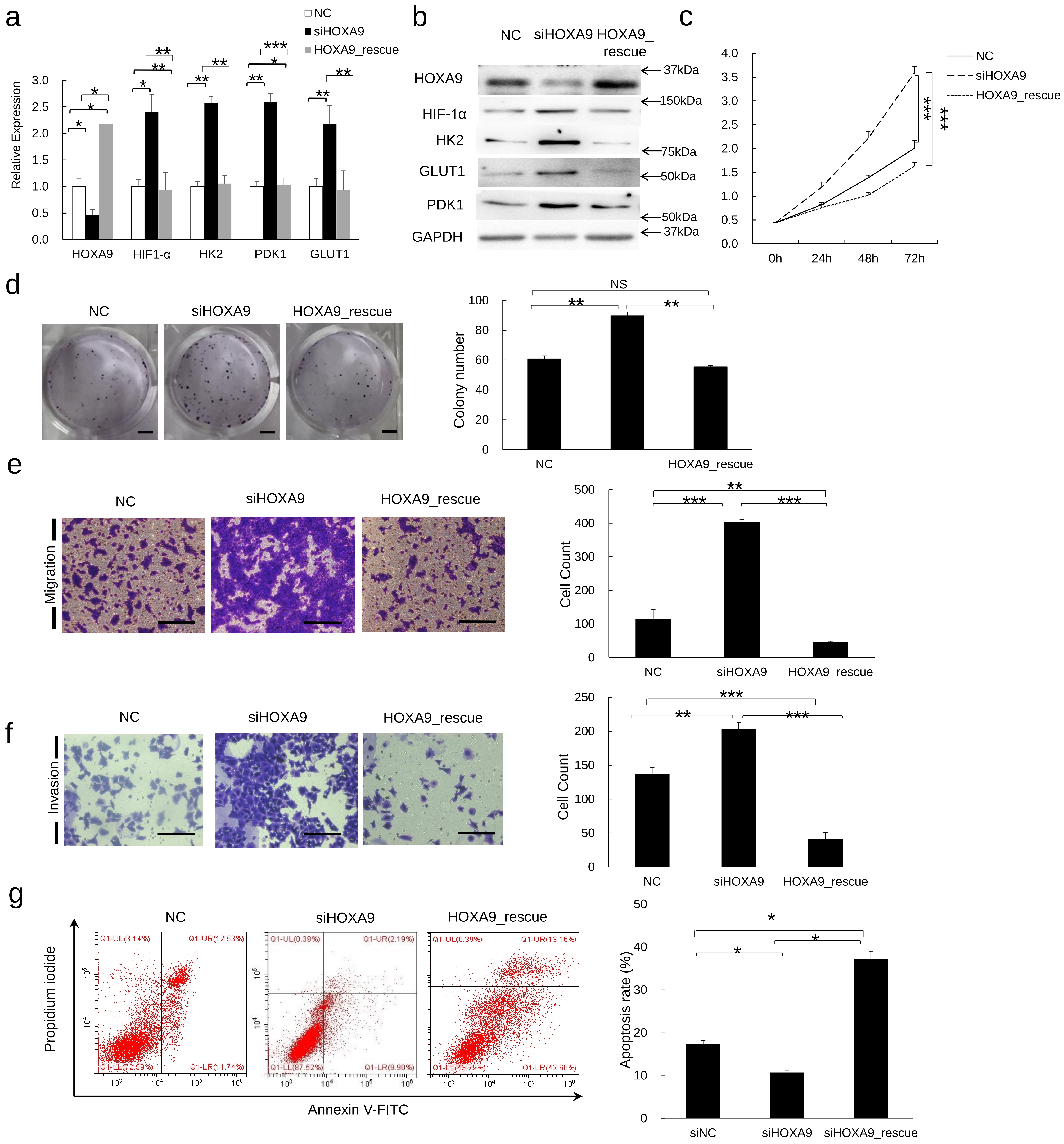

Supplementary Figure 7. Rescue experiments by restoring HOXA9 expression after HOXA9 depletion to verify the tumor-suppressive role of HOXA9. (a,b) HOXA9 mRNA and protein expression was detected by qRT-PCR or western blot. HOXA9 rescue after HOXA9 knockdown re-inhibited the enhanced proliferation (c), colony formation (d), migration (e), invasion (f), and upregulated expression of HIF-1α and its downstream glycolytic genes (a,b) caused by HOXA9 knockdown and re-promoted the apoptosis (g). In (d), scale bar : 1cm. In (e) and (f), scale bar: 100 μm. Each experiment was performed at least in triplicate and data are presented as mean ± s.d. One-Way ANOVA and Dunnett's multiple comparison test were used to analyze the data (*P < 0.05, **P < 0.01, ***P < 0.001).

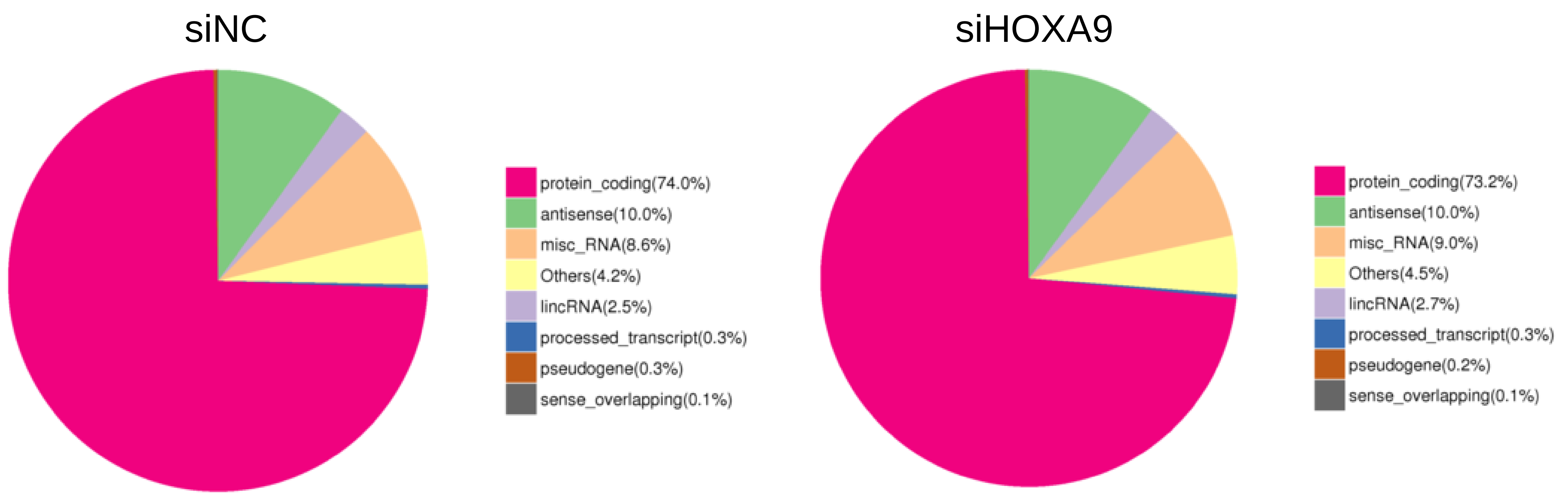

Supplementary Figure 8. Classification of mapped reads from transcriptomic sequencing of siNC- or siHOXA9-treated A431 cells.

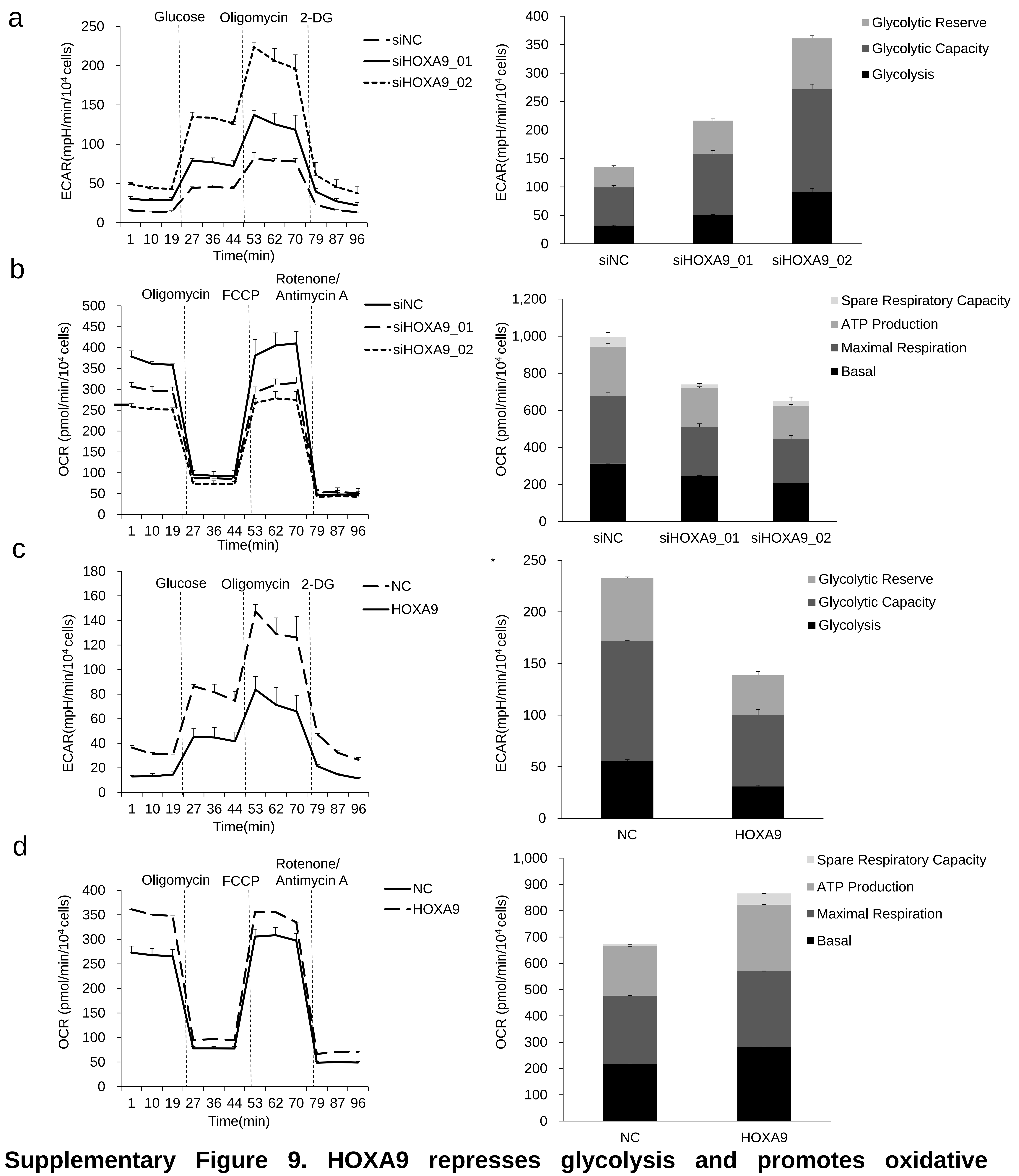

Supplementary Figure 9. HOXA9 represses glycolysis and promotes oxidative phosphorylation in HSC-1 cells. (a-d) Left panels: ECAR and OCR analysis of HSC-1 cells, in which HOXA9 was depleted using two siRNAs or overexpressed, followed by treatment with the indicated compounds; right panels: glycolytic variations (glycolysis, glycolytic capacity, and glycolytic reserve) or OXPHOS variations (basal, maximal respiration, ATP production, and spare respiratory capacity) were summarized from raw data. Each experiment was performed at least in triplicate and data are presented as mean ± s.d. One-Way ANOVA and Dunnett's multiple comparison test were used to analyze the data (**P* < 0.05, ***P* < 0.01, ****P* < 0.001).

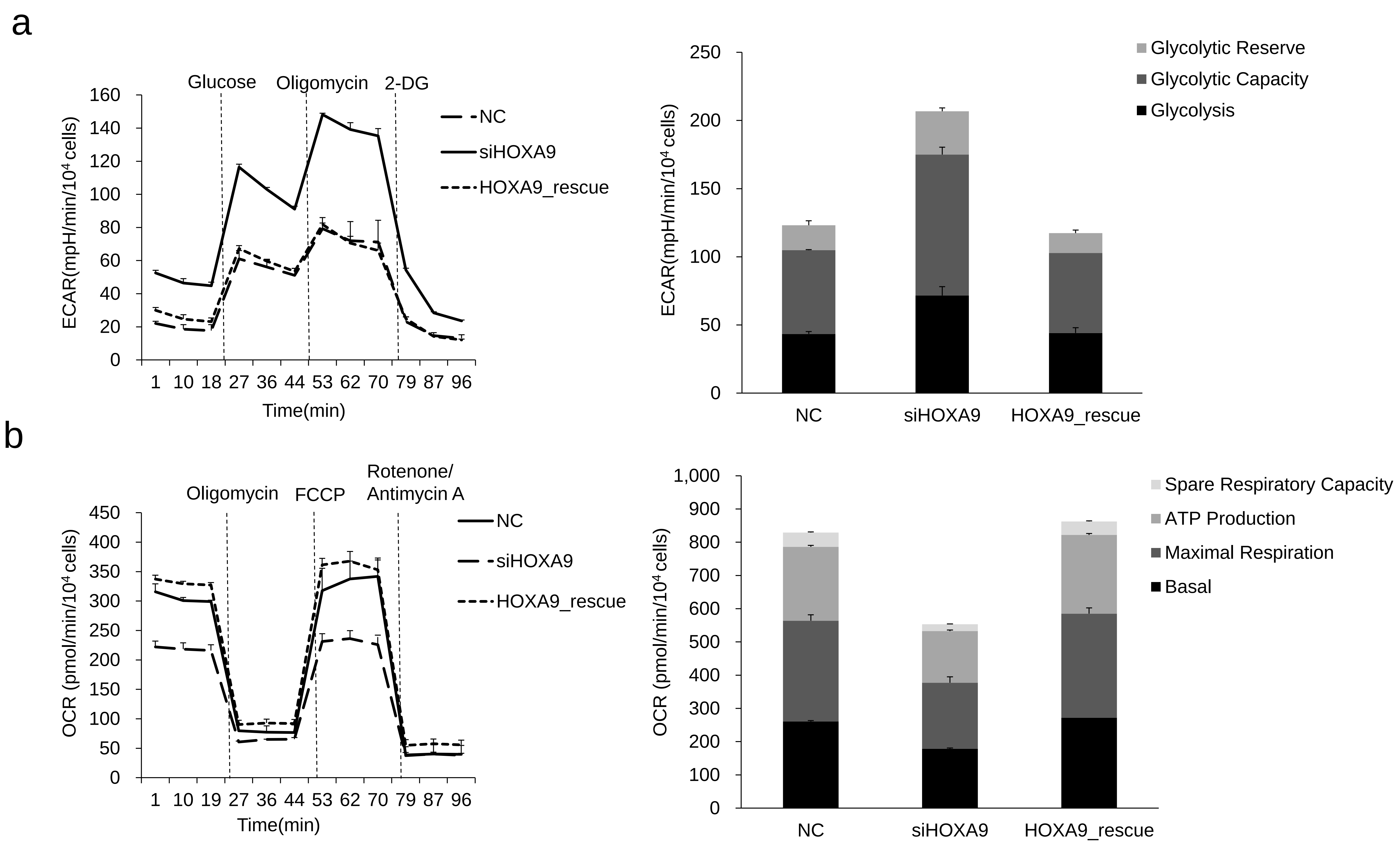

Supplementary Figure 10. Rescue experiments by restoring HOXA9 expression after HOXA9 depletion to verify the glycolysis-inhibitive role of HOXA9. (a,b) Left panels: ECAR and OCR analysis of A431 cells, in which HOXA9 was first depleted by siRNA treatment and then restored by HOXA9-expressing plasmid, followed by treatment with the indicated compounds; right panels: glycolytic variations (glycolysis, glycolytic capacity, and glycolytic reserve) or OXPHOS variations (basal, maximal respiration, ATP production, and spare respiratory capacity) were summarized from raw data. Each experiment was performed at least in triplicate and data are presented as mean ± s.d. One-Way ANOVA and Dunnett's multiple comparison test were used to analyze the data (*$P < 0.05$, **$P < 0.01$, ***$P < 0.001$).

CRIP2 peptide A: ASSVTTFTGEPNTCPR. t = 30.46min

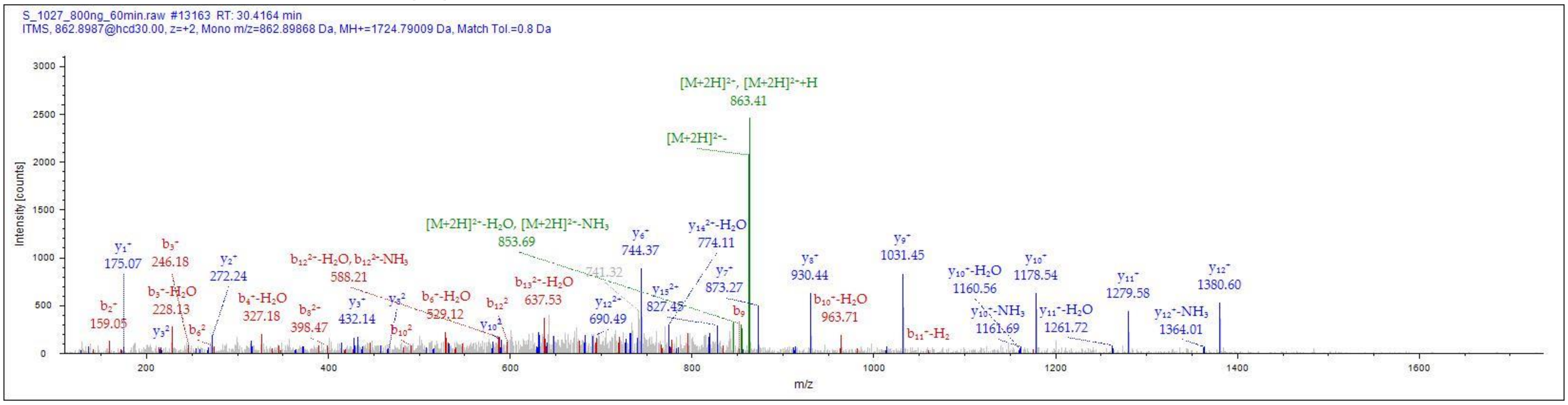

CRIP2 peptide B: GVNIGGAGSYIYEKPLAEGPQVTGPIEVPAAR. t = 51.21min

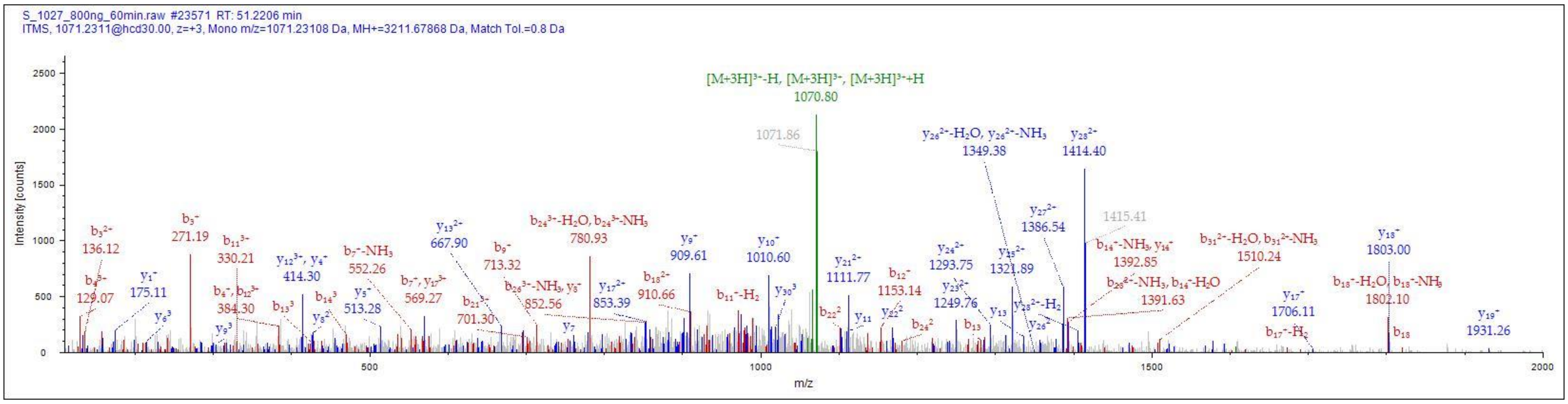

Supplementary Figure 11. The amino acid sequences of two CRIP2 peptides were identified by high performance liquid chromatography-mass spectrometry (HPLC-MS) analysis of the protein mix immunoprecipitated by HOXA9 antibody.

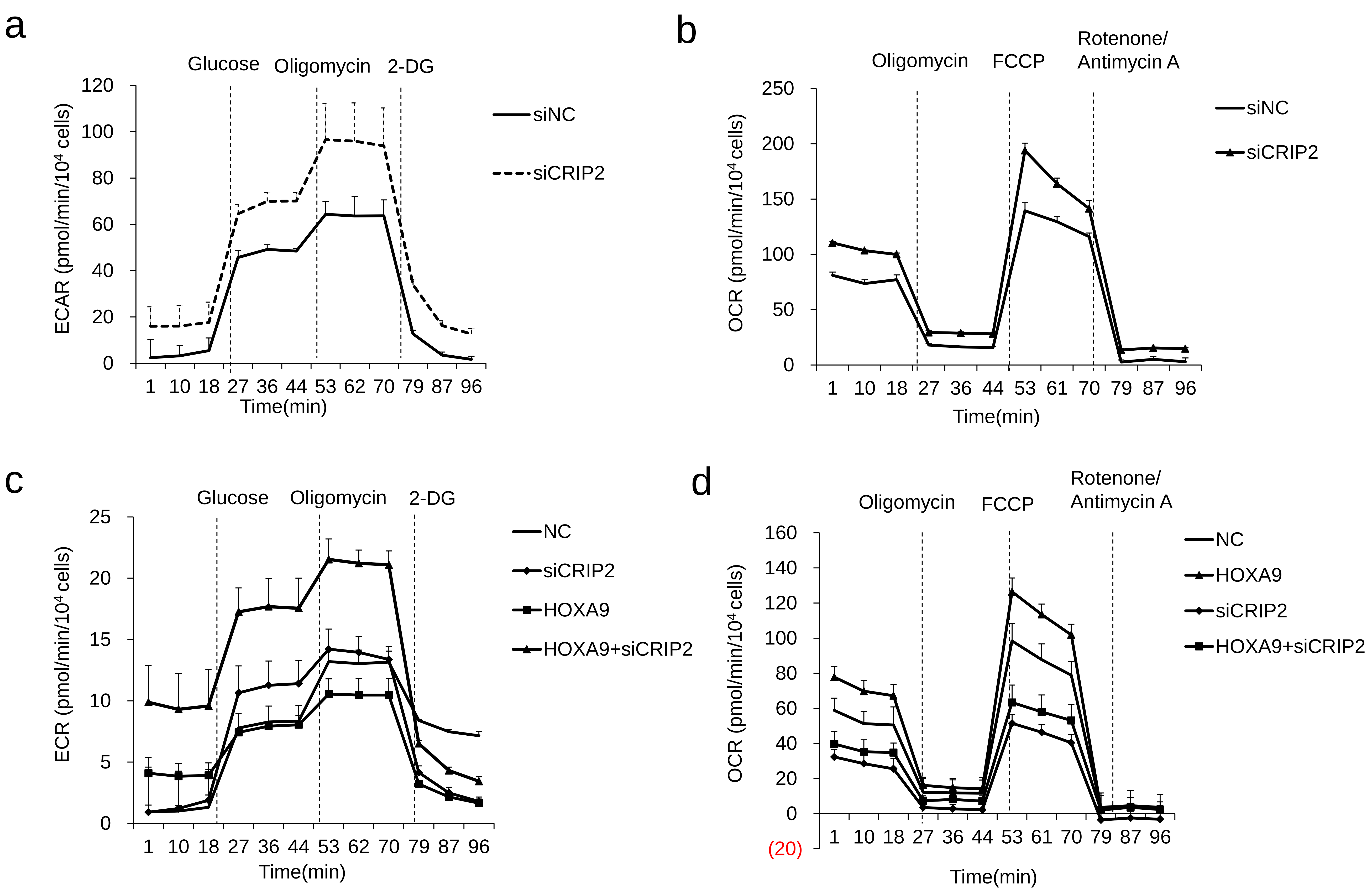

Supplementary Figure 12. The role of CRIP2 in glucose metabolism of CSCC was evaluated by OCR and ECAR assay. (a,b) Left panels: ECAR and OCR analysis of A431 cells, in which CRIP2 was depleted using siRNA or HOXA9 was overexpressed by HOXA9-expressing plasmid as indicated, followed by treatment with the indicated compounds; right panels: glycolytic variations (glycolysis, glycolytic capacity, and glycolytic reserve) or OXPHOS variations (basal, maximal respiration, ATP production, and spare respiratory capacity) were summarized from raw data. Each experiment was performed at least in triplicate and data are presented as mean ± s.d. One-Way ANOVA and Dunnett's multiple comparison test were used to analyze the data (**P* < 0.05, ***P* < 0.01, ****P* < 0.001).

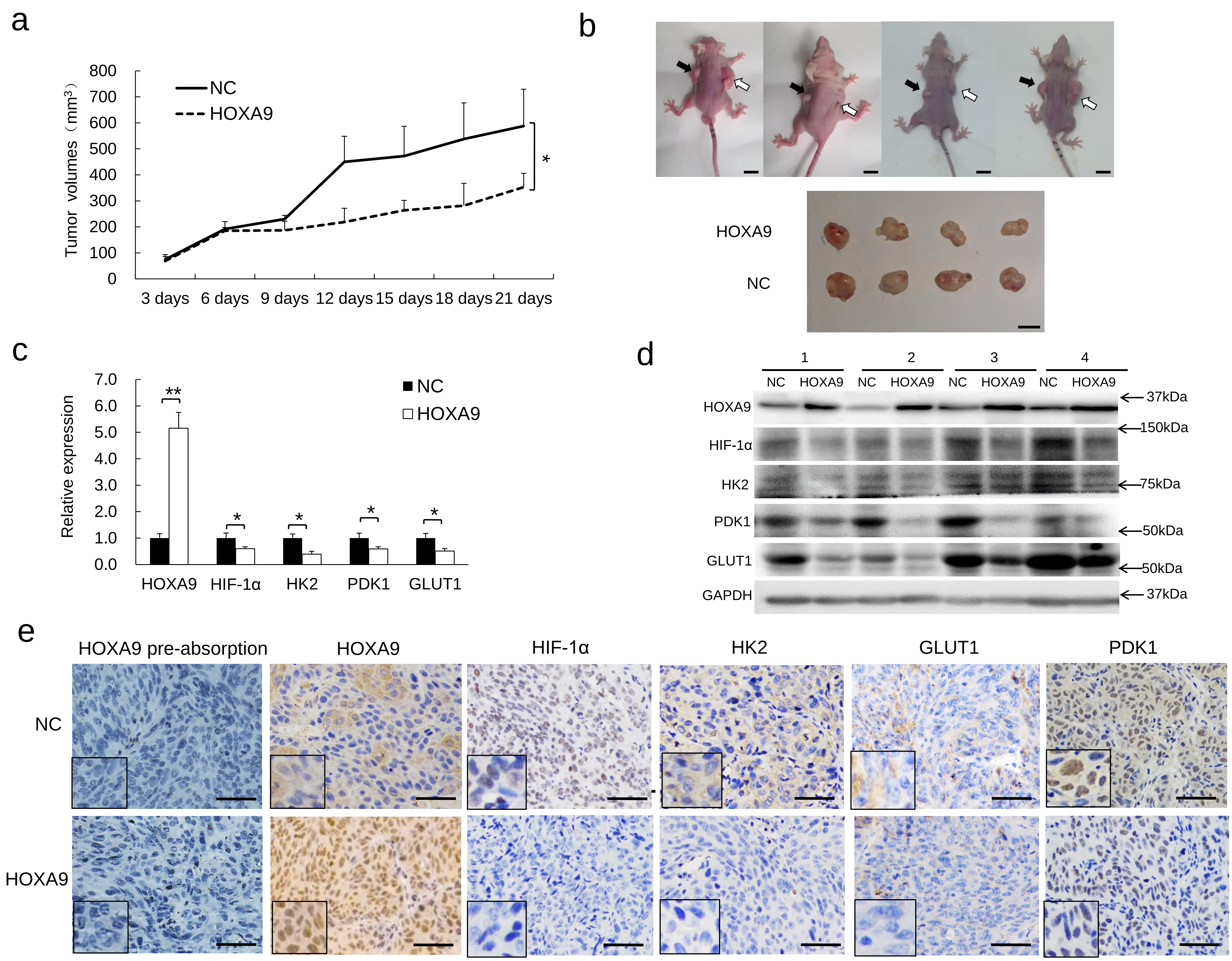

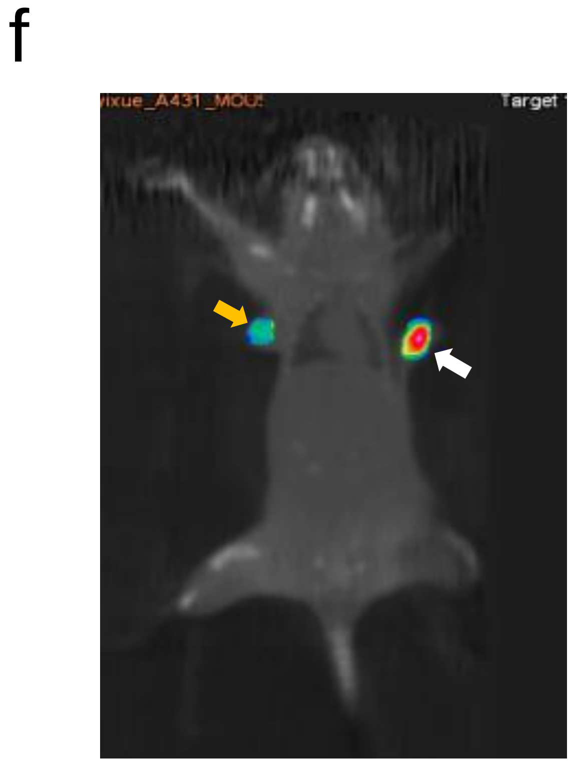

Supplementary Figure 13. HOXA9 overexpression represses glycolysis and tumor growth in *vivo*. Empty vector or HOXA9 expressing vector were injected into A431 cell xenografts every three days. (a) Overexpression of HOXA9 inhibits subcutaneous tumor growth in a mouse xenograft model. Tumor volumes (mm^3) were plotted according to day. (b) The mice were sacrificed at the end of the experimental period and images taken along with the dissected tumors from four representative mice are shown. White arrows indicate the empty vector-treated xenografts whereas black arrows indicate HOXA9 expressing vector-treated xenografts. Scale bar, 1cm.(c) The expression of *HOXA9, HIF1A, HK2, GLUT1*, and *PDK1* was measured in the dissected tumors by qRT-PCR. (d) The protein expression of HOXA9, HIF-1α, HK2, GLUT1, and PDK1 was detected in xenografts after HOXA9 overexpression by using western blot. (e) Histopathology analysis (IHC staining) of HOXA9, HIF-1α, HK2, GLUT1, and PDK1 on tumor sections. The quantification was performed by counting positively stained cells from 10 randomly chosen fields from a total of five sections per tumor. Scale bar, 100 μm. (f) Comparison of glucose consumption between empty vector- and HOXA9 expressing vector-treated xenograft tumors by microPET/CT imaging of the uptake and retention of ^{18}F-FDG administered by tail vein injection. Representative microPET/CT image is shown. Each experiment was performed at least in triplicate and data are presented as mean ± s.d. One-Way ANOVA and Dunnett's multiple comparison test were used to analyze the data (*P < 0.05, **P < 0.01, ***P < 0.001).

HaCaT cell line

STR Profile:

STR Profile	AMEL	CSF1PO	D13S317	D16S539	D5S818	D7S820	TH01	TPOX	vWA
HaCaT	X	9 11	10 12	9 12	12	9 11	9.3	11 12	16 17

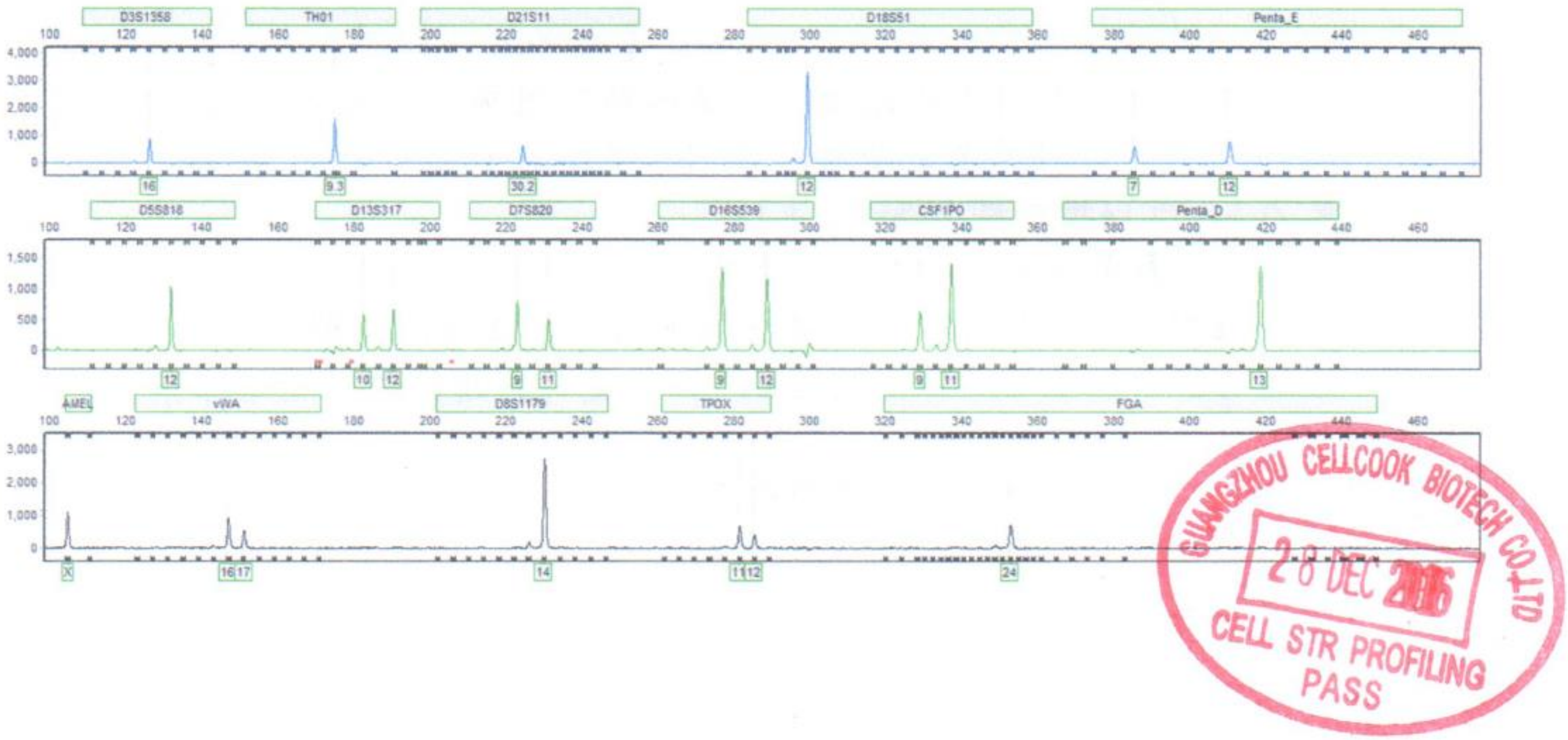

A431 cell line

STR 位点信息:

STR Profile	AMEL	CSF1PO	D13S317	D16S539	D5S818	D7S820	TH01	TPOX	vWA
A431	X	11, 12	9, 13	12, 14	12, 13	10	9	11	15, 17

STR 鉴定图谱:

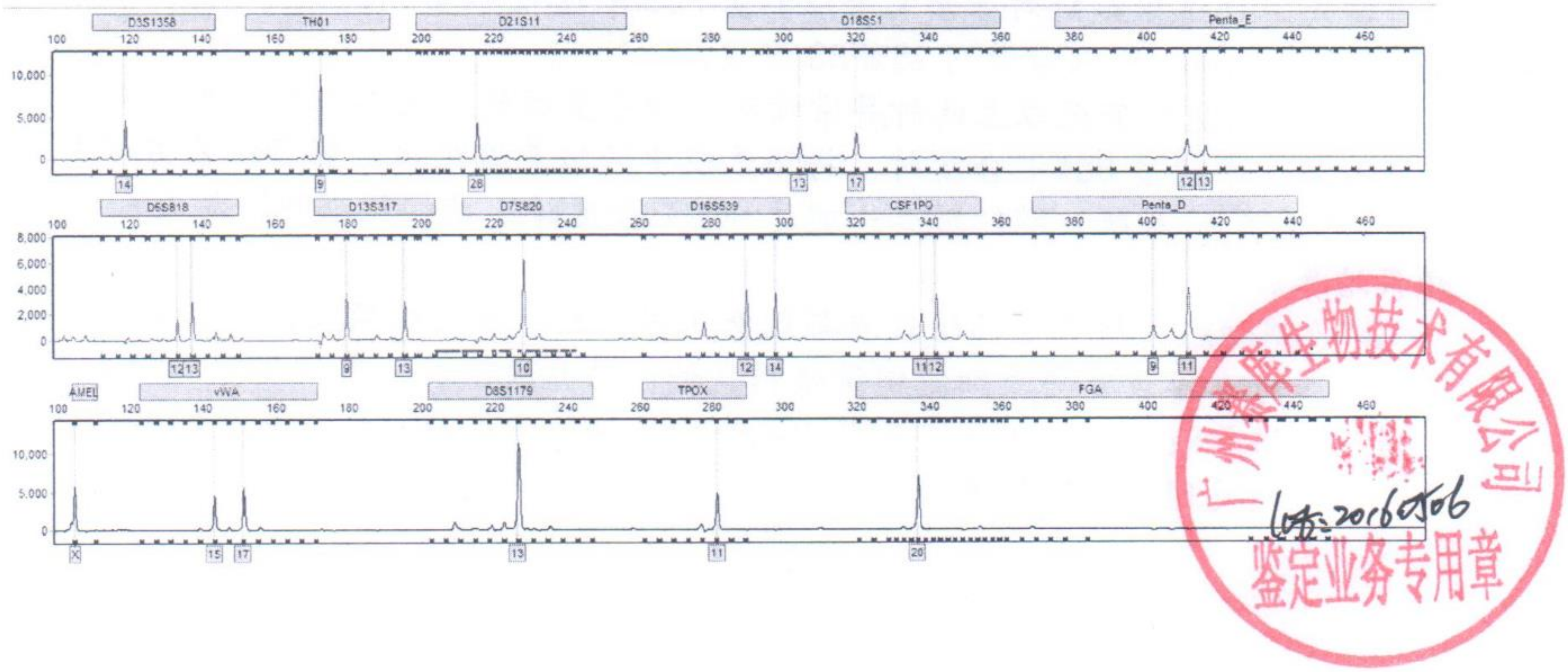

Supplementary Fig. 14. Authentication of cell lines by STR typing.

HSC-1 cell line

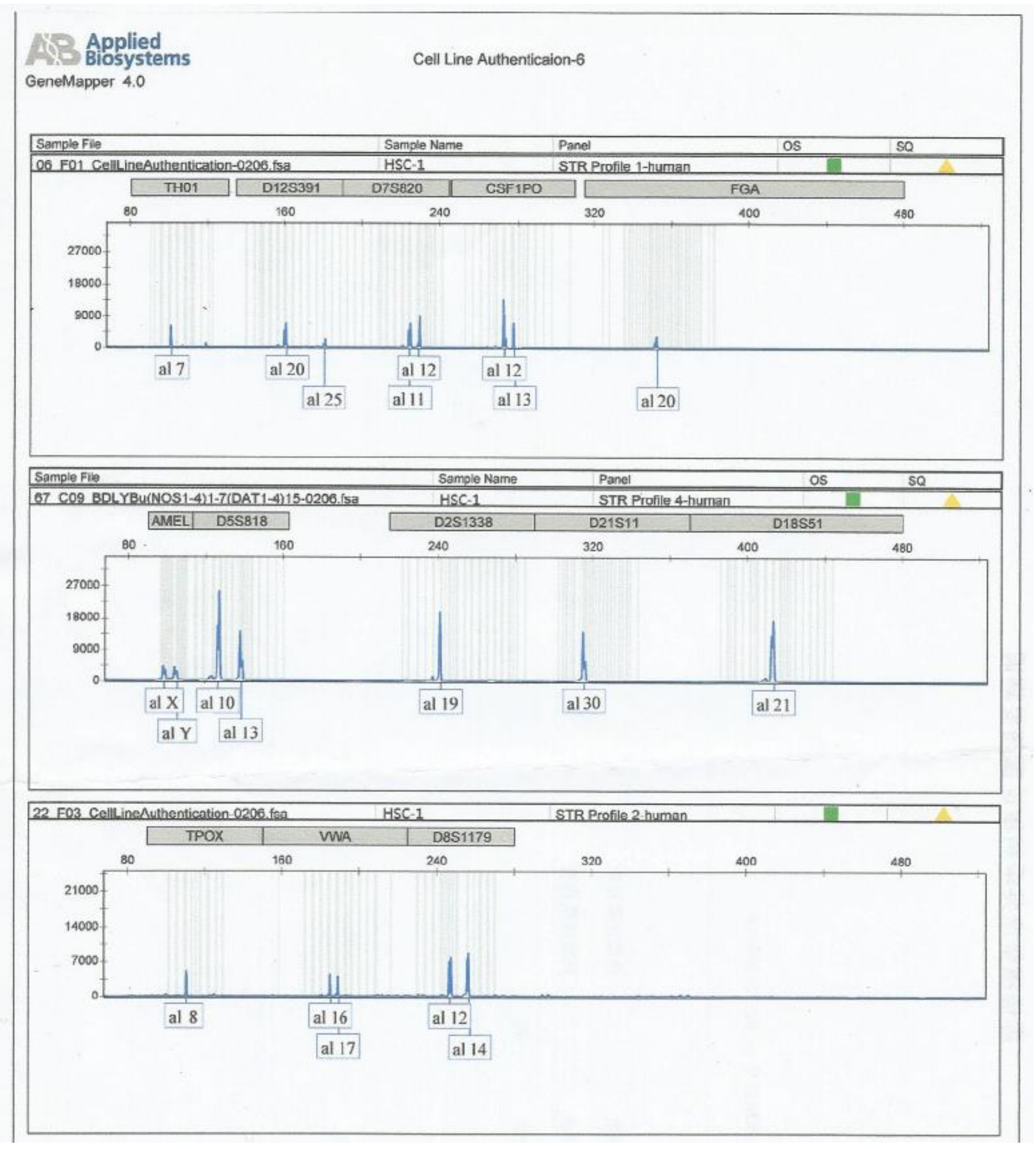

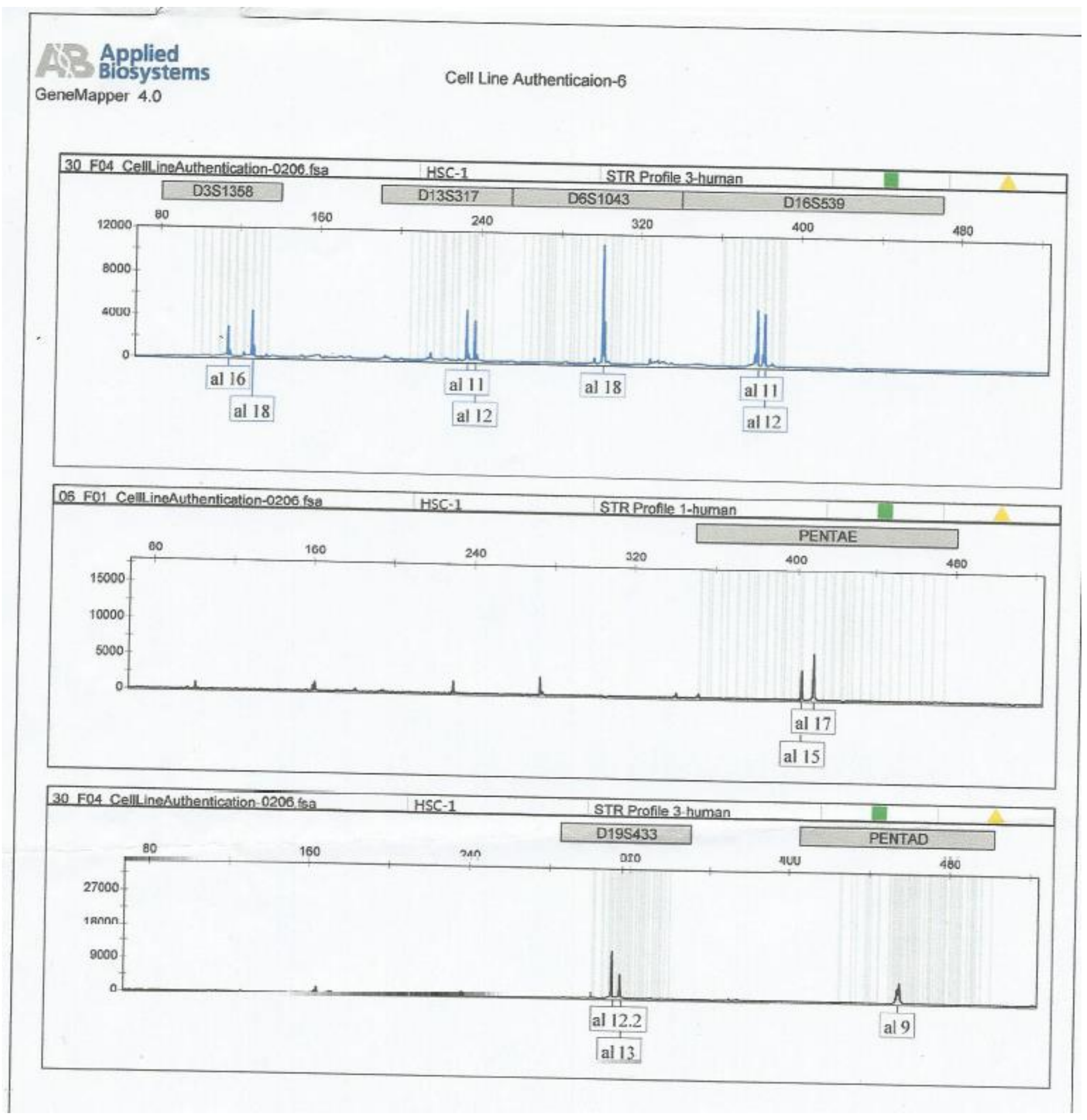

遗传质量鉴定检验报告

样品编号：

表 1 样本编号

客户样本编号	公司编号
HSC-1	20170206-01

样品数量： 1

样品性状： 细胞系

检测项目： STR

送检单位： 弘顺生物

检测方法： 用 Axygen 的基因组抽提试剂盒提取 DNA，采用 20- STR 扩增方案扩增，在 ABI 3730XL 型遗传分析仪上对 STR 位点和性别基因 Amelogenin 进行检测。

上海翼和应用生物技术有限公司

检验结果：

(一)检验基本情况

表 2：样本基因型检验结果

样本编号	多等位基因	匹配细胞系	细胞库	EV 值	匹配说明
20170206-01	无	HSC-1	JCRB	1	完全匹配

- 多等位基因指三等位及以上基因现象。
- 本次检测各细胞分型结果良好。

(二)各样本描述

- 20170206-01：该株细胞 DNA 分型在细胞系检索中找到完全匹配的细胞系，JCRB 数据库显示细胞名为 **HSC-1**，细胞号对应 **JCRB1015**。本次检测在该细胞系中**没有发现多等位基因**。
- **备注：** 待测细胞系与收录于 ATCC, DSMZ, JCRB 和 RIKEN 数据库的细胞系 STR 数据进行比对，未收录于以上细胞库的细胞系将无法匹配。

(三)样本分型结果

表 3：细胞 20170206-01 的 STR 位点和 Amelogenin 位点的基因分型结果

Marker	样本				细胞库信息		
	Allele1	Allele2	Allele3	Allele4	Allele1	Allele2	Allele3
D5S818	10	13			10	13	
D13S317	11	12			11	12	
D7S820	11	12			11	12	
D16S539	11	12			11	12	
VWA	16	17			16	17	
TH01	7	7			7	7	
AMEL	X	Y			X	Y	
TPOX	8	8			8	8	
CSF1PO	12	13			12	13	
D12S391	20	25					
FGA	20	20					
D2S1338	19	19					
D21S11	30	30					
D18S51	21	21					
D8S1179	12	14					
D3S1358	16	18					
D6S1043	18	18					
PENTAE	15	17					
D19S433	12.2	13					
PENTAD	9	9					

其他说明：

(一)分型方案及位点分布：

上海翼和应用生物技术有限公司

附表：实验方案及位点

Supplementary Fig. 14 (continued). Authentication of cell lines by STR typing.

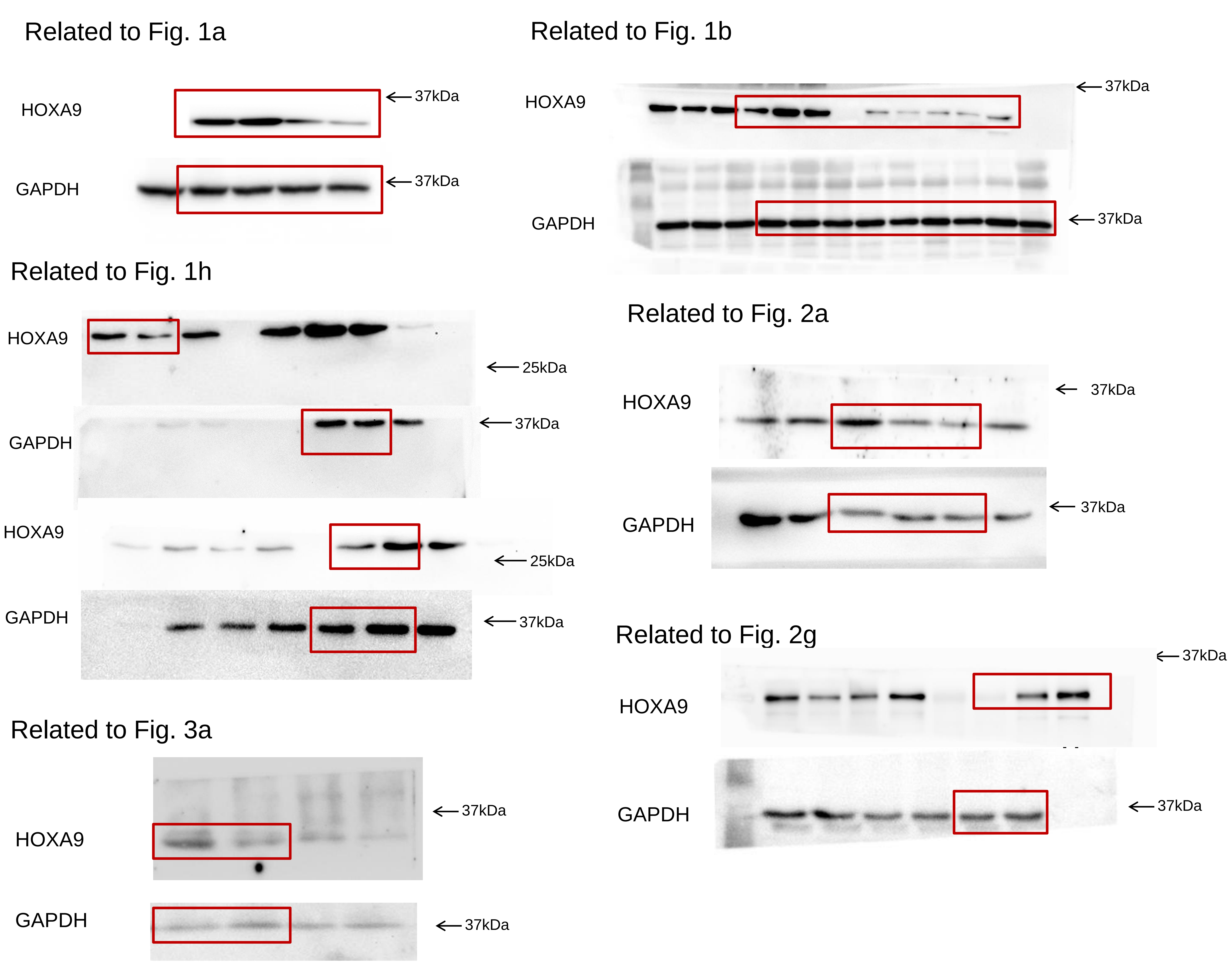

Supplementary Figure 15. Uncropped images of blots and gels with size markers are presented. Reference to the original figures is presented for each blot or gel.

Related to Fig. 5b

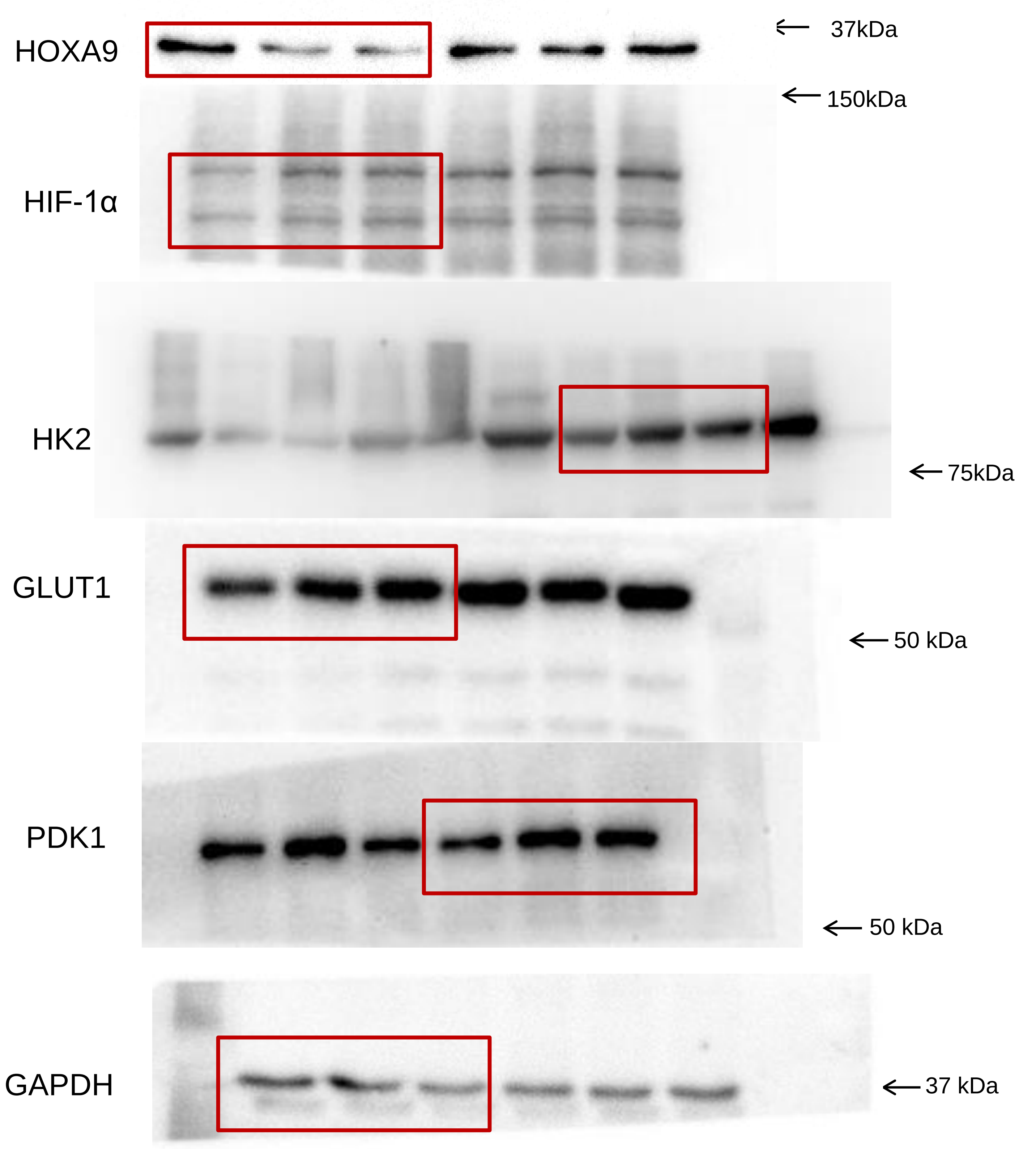

Related to Fig. 5b

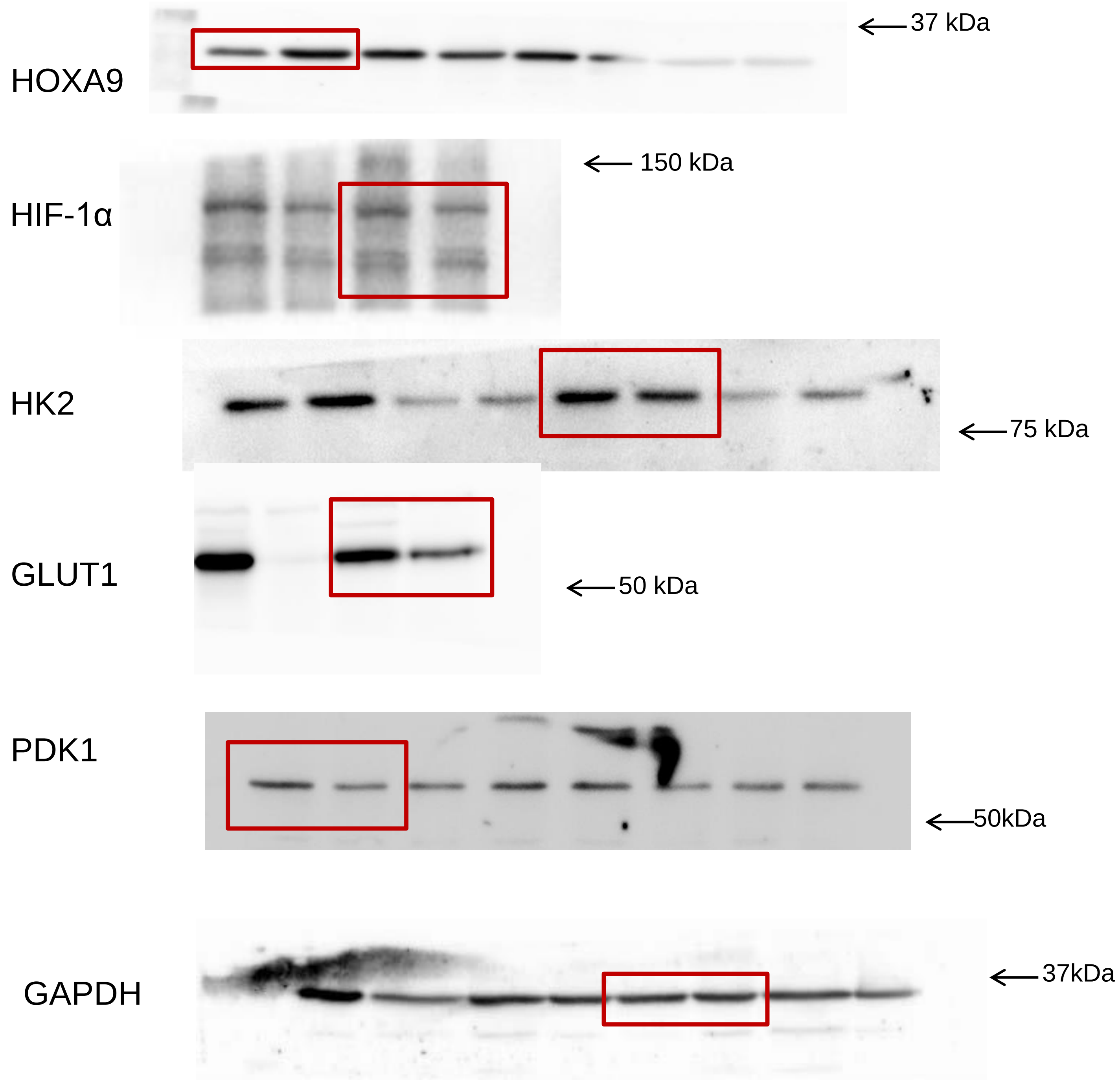

Supplementary Figure 15 (continued). Uncropped images of blots and gels with size markers are presented. Reference to the original figures is presented for each blot or gel.

Related to Fig. 5d

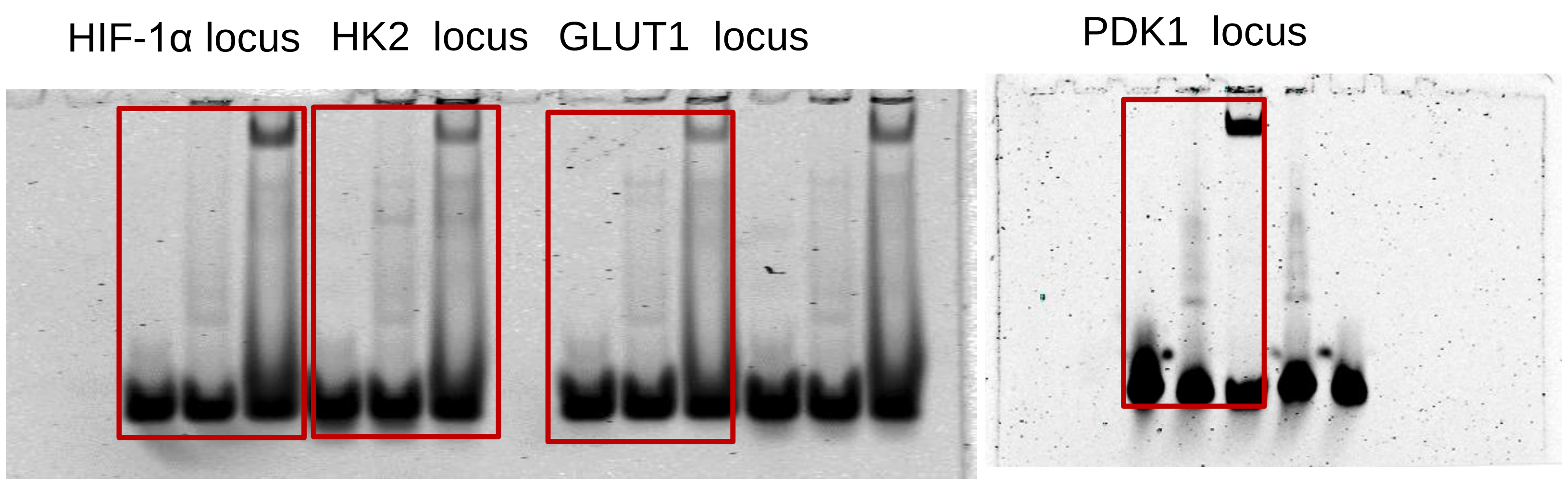

Probe mutant

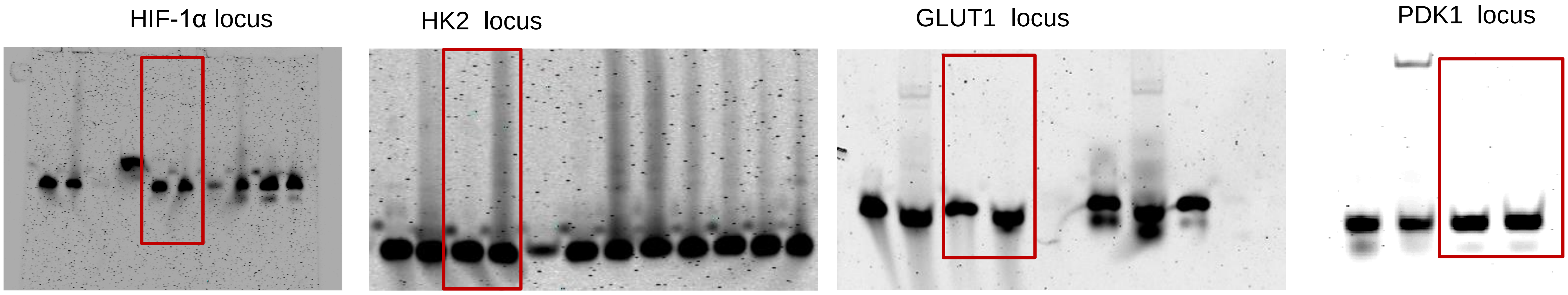

Related to Fig. 6b

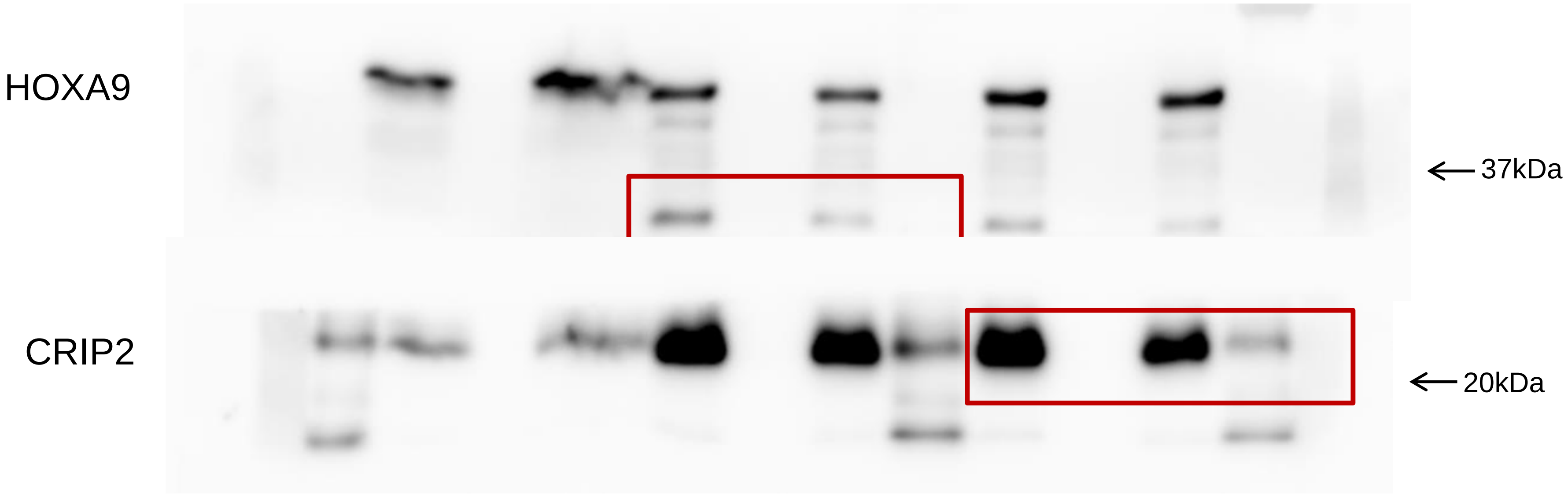

Related to Fig. 6c

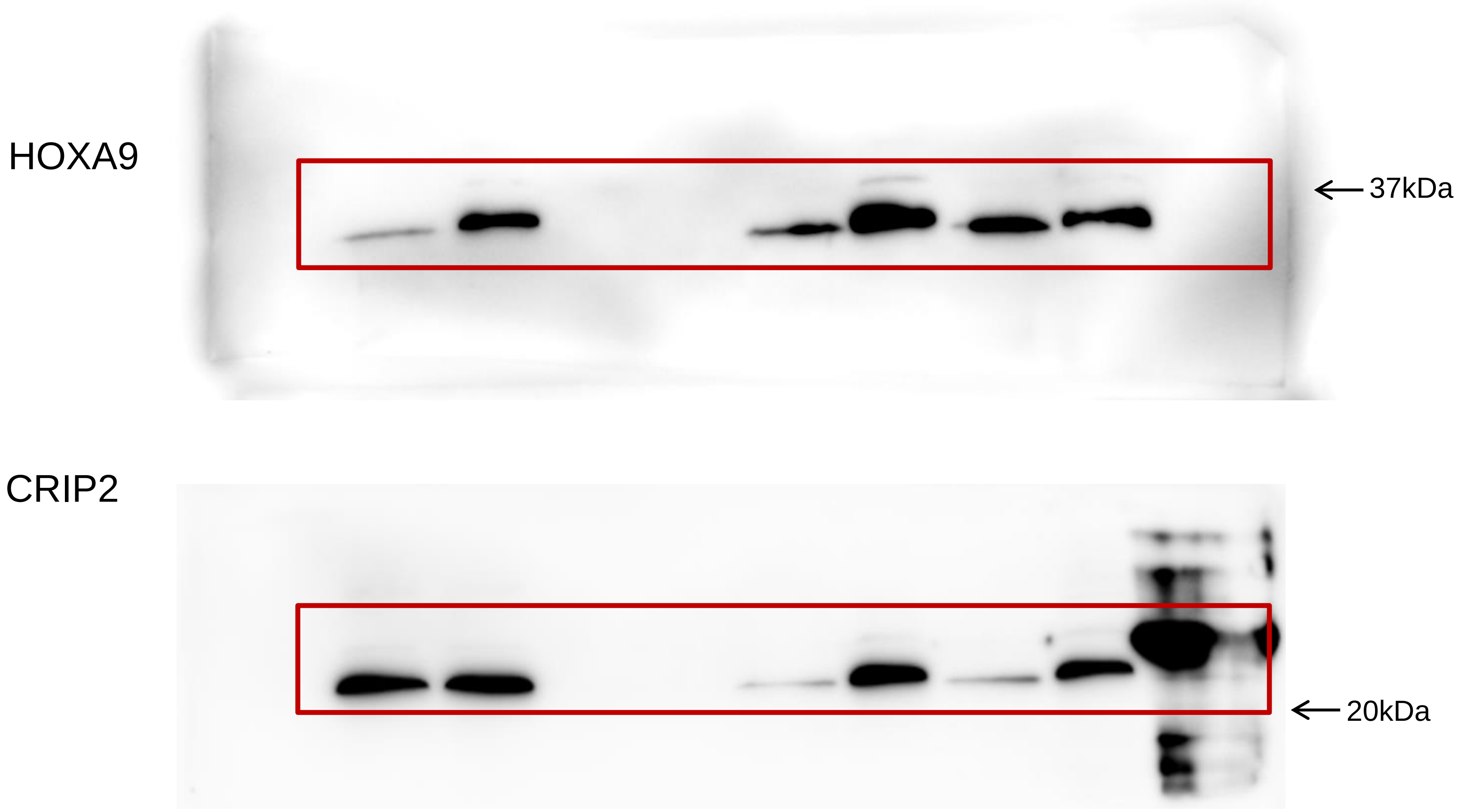

Supplementary Figure 15 (continued). Uncropped images of blots and gels with size markers are presented. Reference to the original figures is presented for each blot or gel.

Related to Fig. 7a

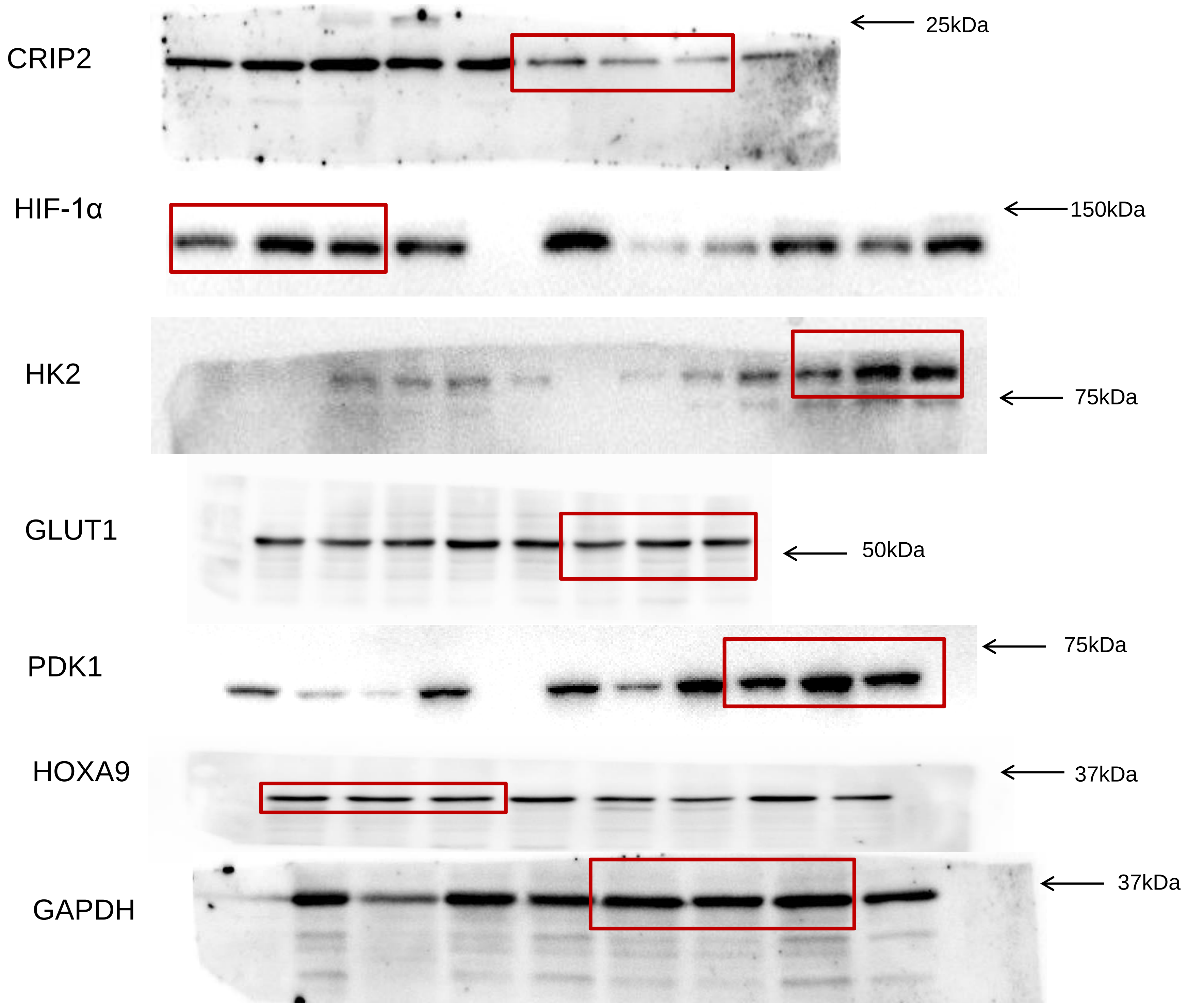

Related to Fig. 7g

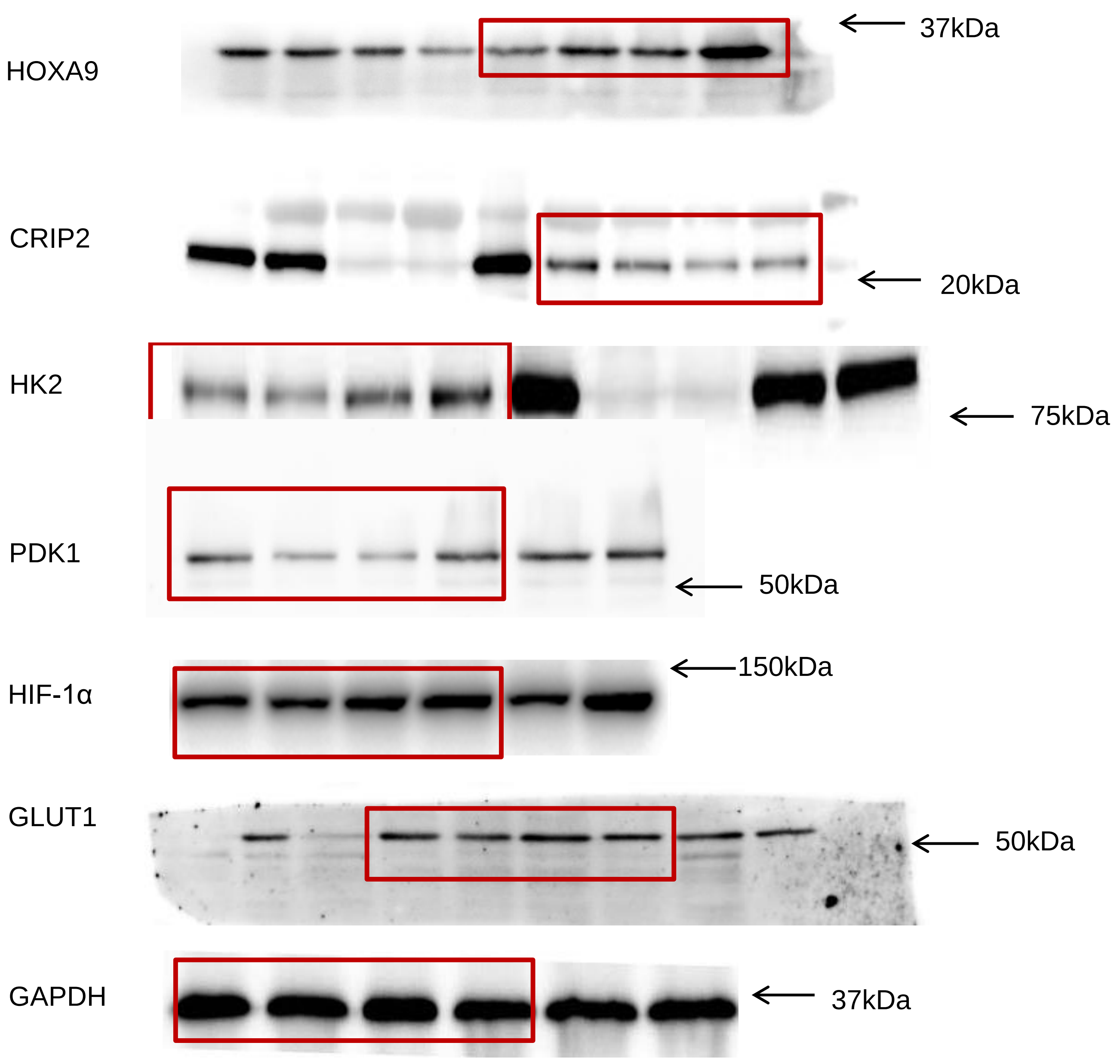

Supplementary Figure 15 (continued). Uncropped images of blots and gels with size markers are presented. Reference to the original figures is presented for each blot or gel.

Related to Fig. 8d

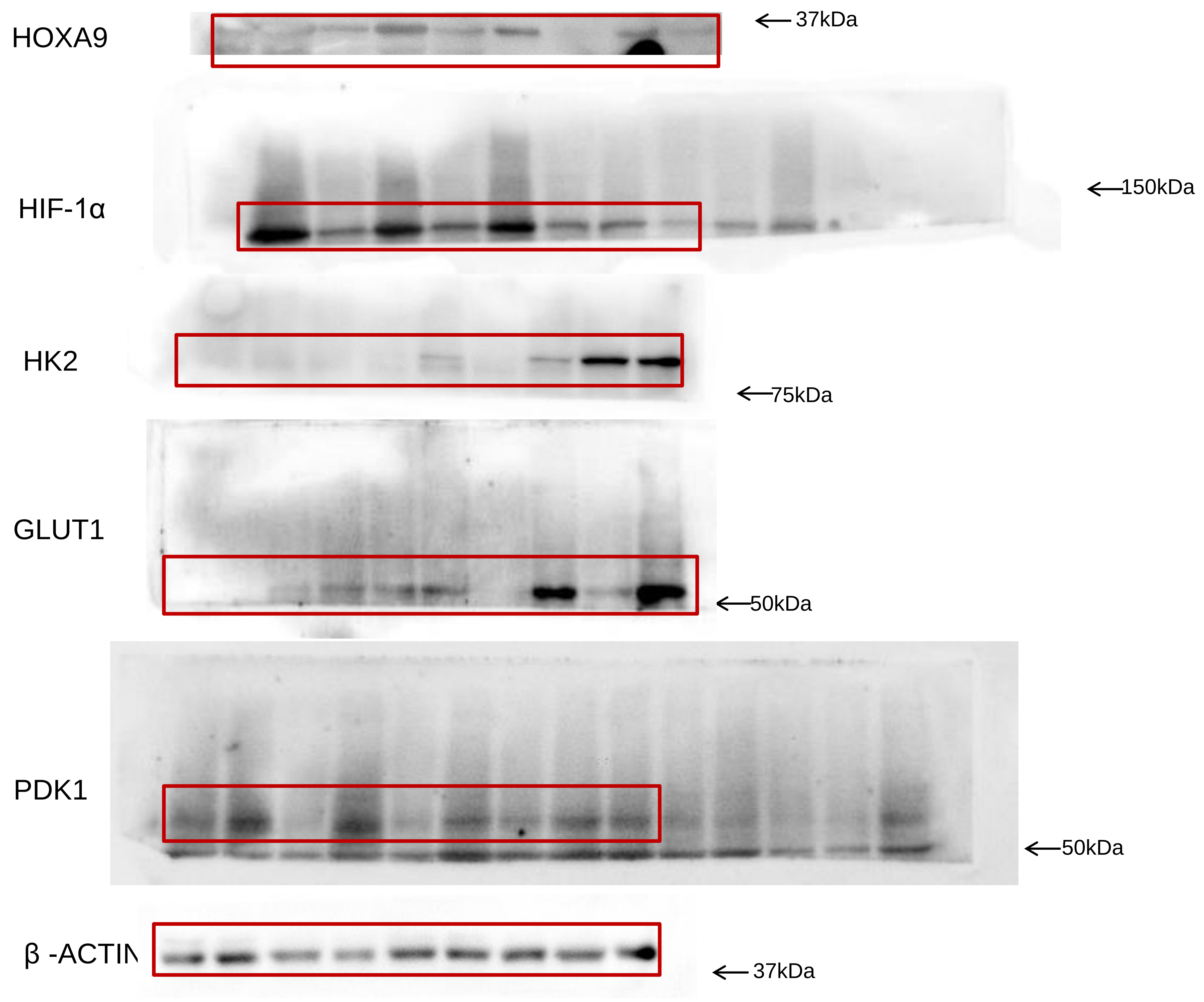

Related to Supplementary Fig. 5b

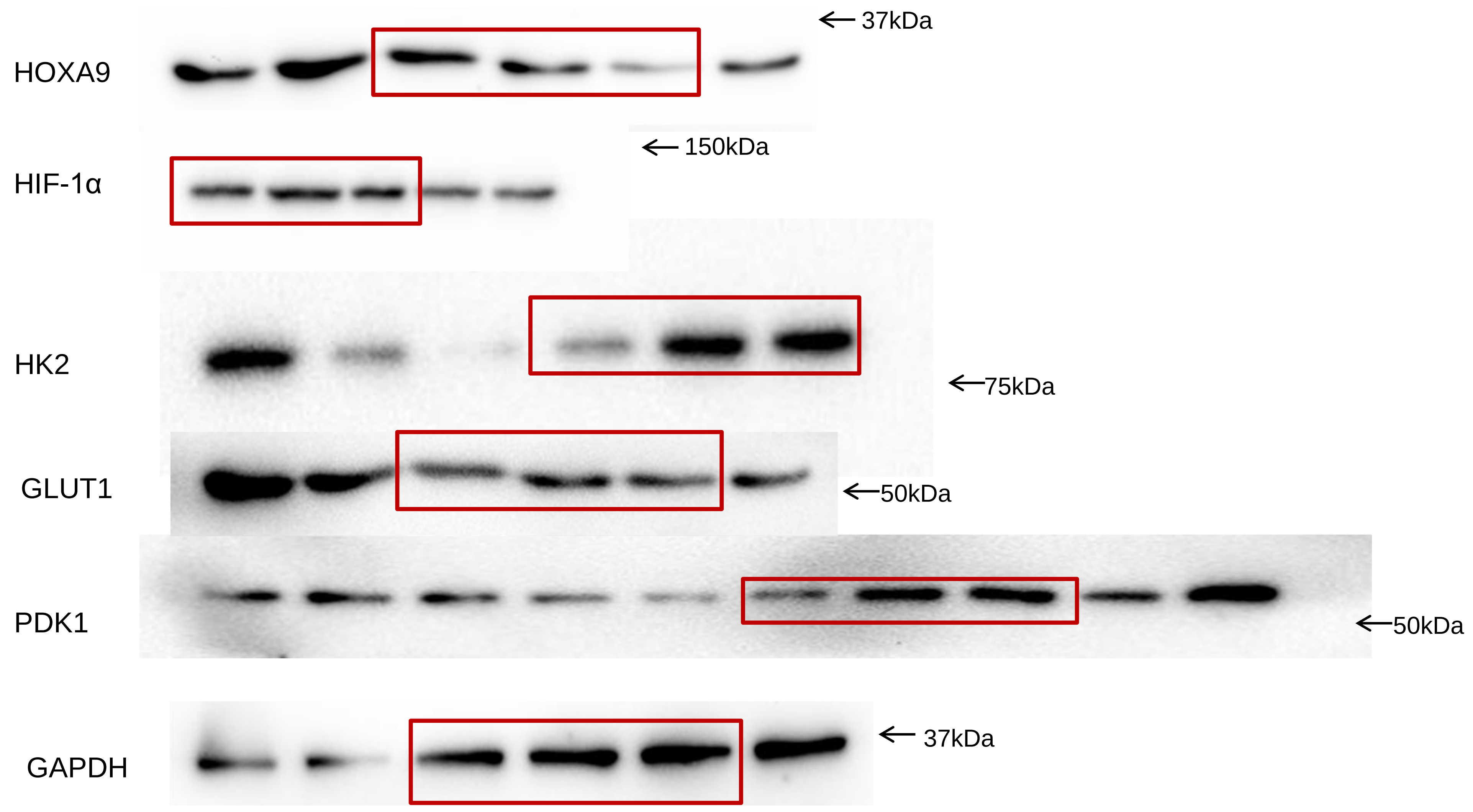

Supplementary Figure 15 (continued). Uncropped images of blots and gels with size markers are presented. Reference to the original figures is presented for each blot or gel.

Related to Supplementary Fig. 6b

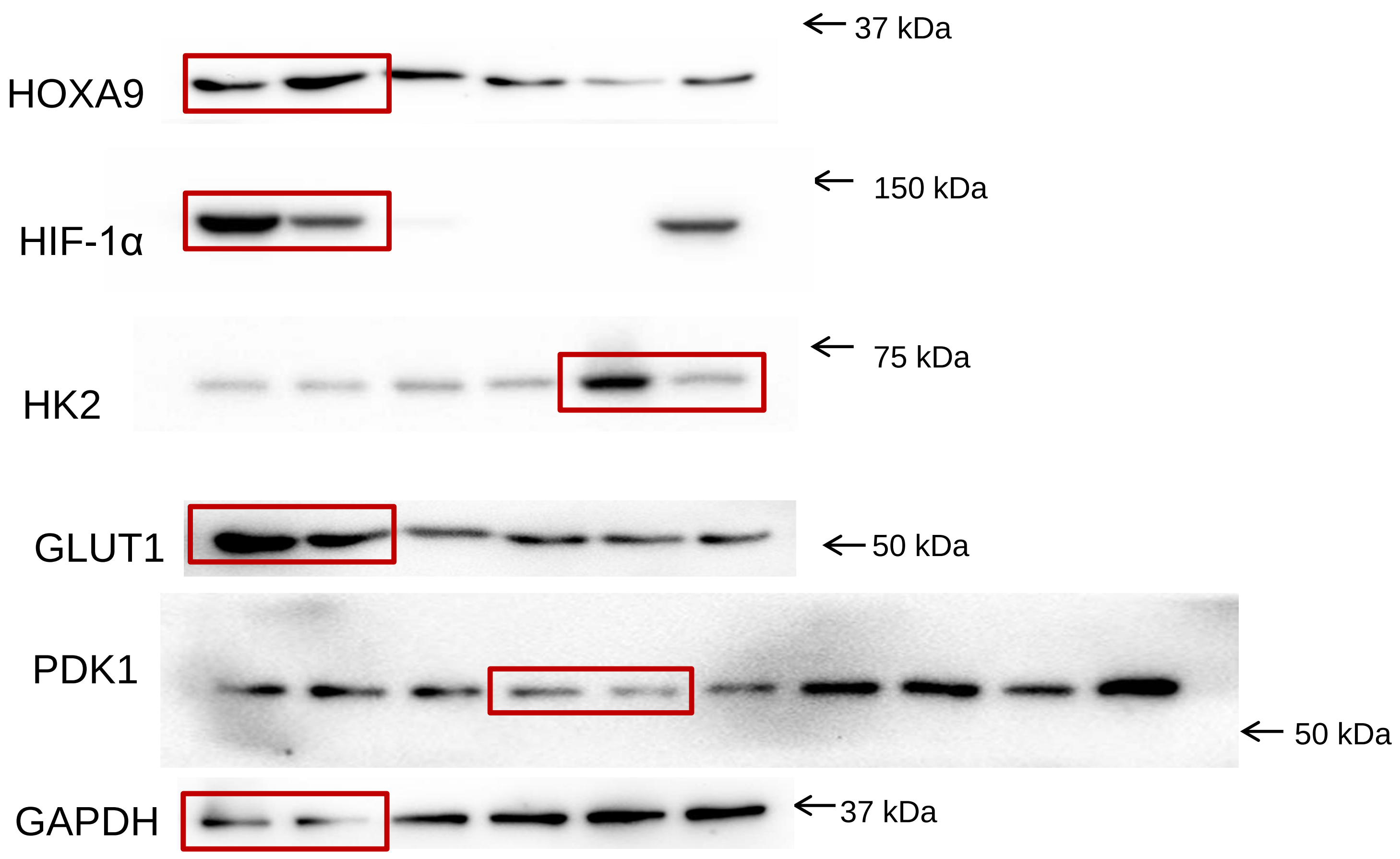

Related to Supplementary Fig. 7b

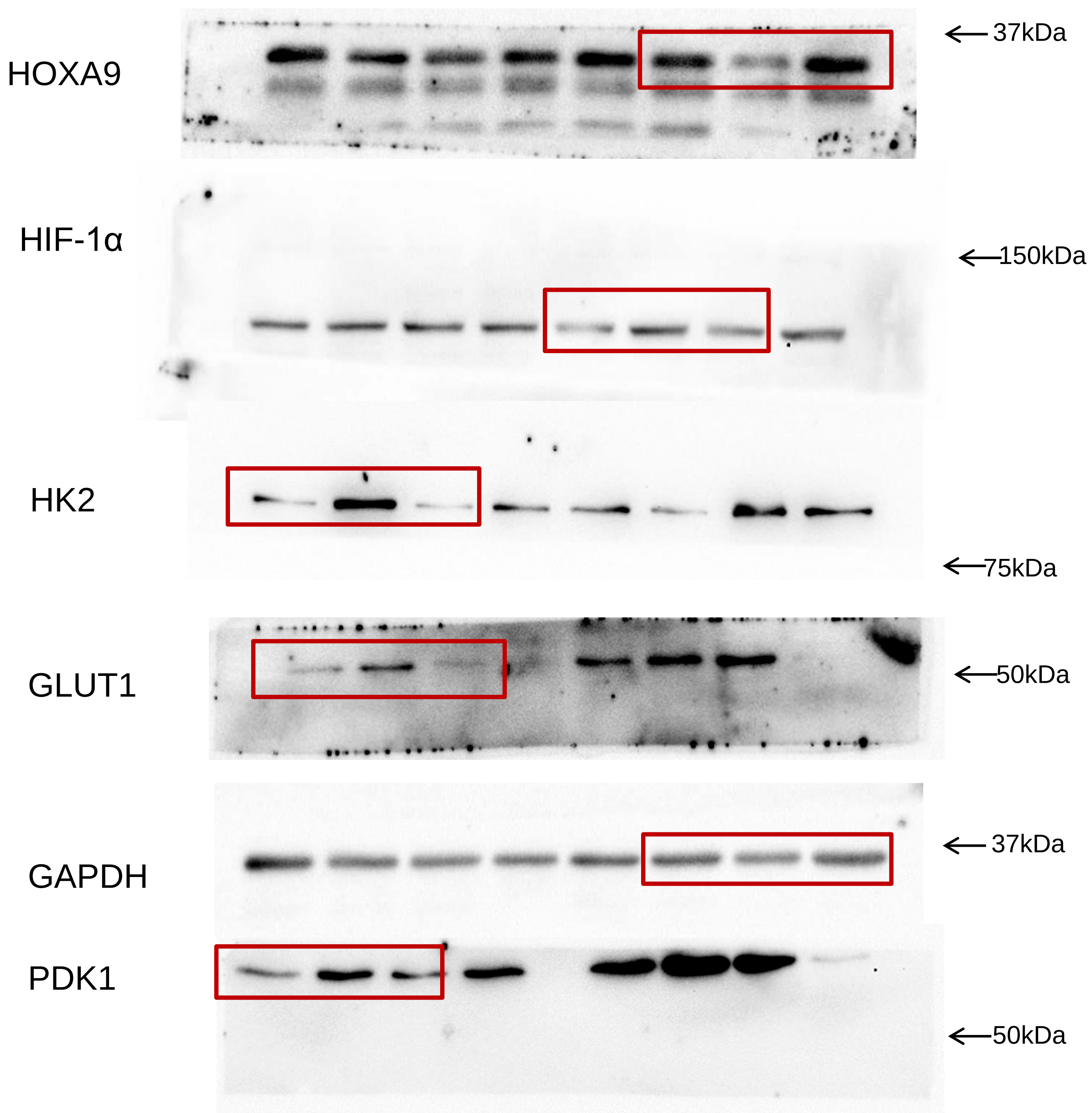

Supplementary Figure 15 (continued). Uncropped images of blots and gels with size markers are presented. Reference to the original figures is presented for each blot or gel.

Related to Supplementary Fig. 13d

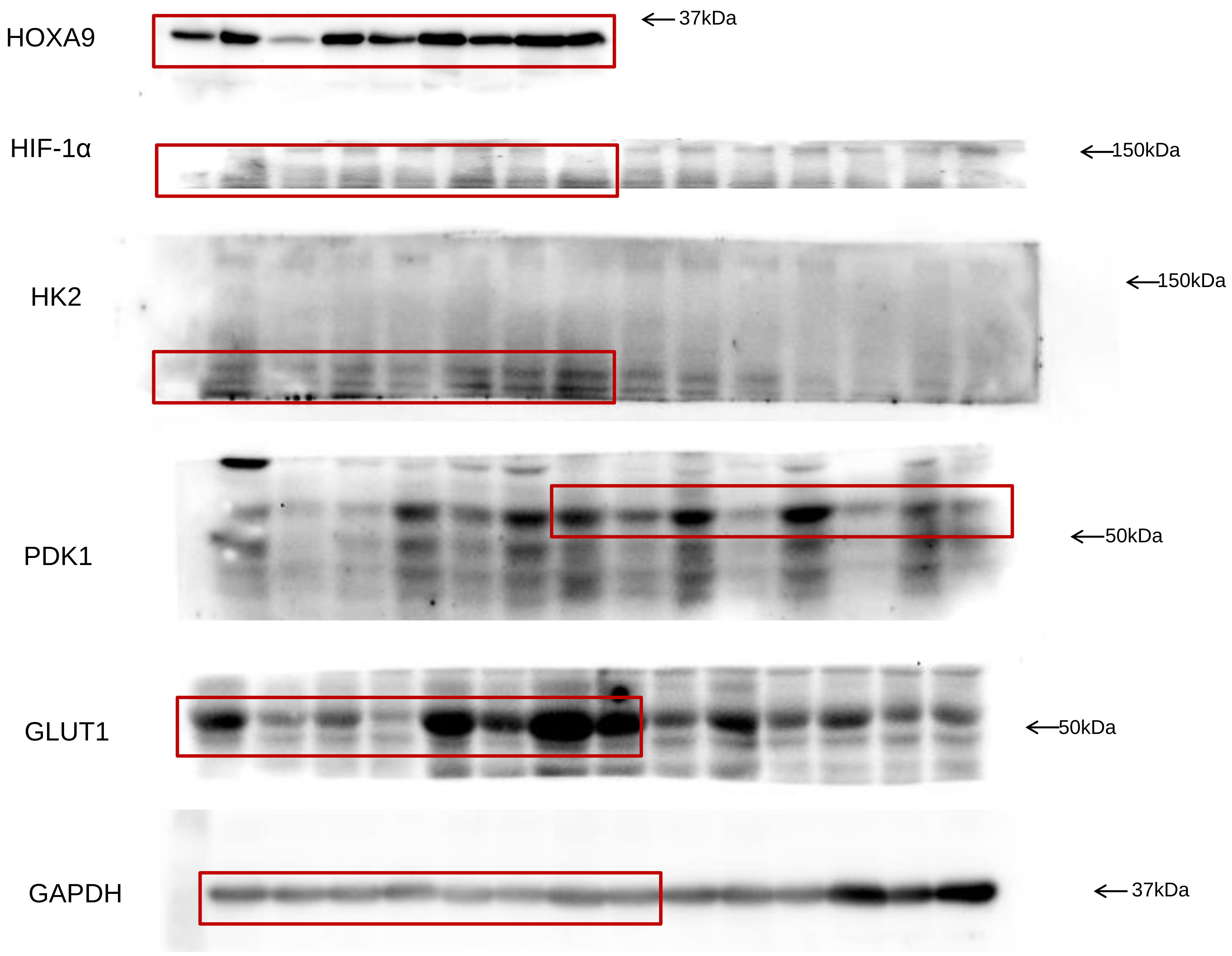

Supplementary Figure 15 (continued). Uncropped images of blots and gels with size markers are presented. Reference to the original figures is presented for each blot or gel.

Supplementary Table 1 Co-IP protein list of HOXA9 identified by mass spectrometry

Accession	Gene name	Description	IgG	HOXA9
Q15323	KRT31	Keratin, type I cuticular Ha1	-	+
Q14525	KRT33B	Keratin, type I cuticular Ha3-II	-	+
O76009	KRT33A	Keratin, type I cuticular Ha3-I	-	+
O43790	KRT86	Keratin, type II cuticular Hb6	-	+
P78386	KRT85	Keratin, type II cuticular Hb5	-	+
P68431	HIST1H3C	Histone H3.1	-	+
P84243	H3F3A	histone H3.3	-	+
P01876	IGHA1	Ig alpha-1 chain C region	-	+
P01860	IGHG3	Ig gamma-3 chain C region	-	+
P35579-1	MYH9	Myosin-9	-	+
Q7Z3E1	TIPARP	TCDD-inducible poly [ADP-ribose] polymerase	-	+
P61626	LYZ	lysozyme c	-	+
O43426	SYNJ1	Synaptojanin-1	-	+
P58107	EPPK1	epiplakin	-	+
Q8N841-1	TTLL6	Tubulin polyglutamylase ttll6	-	+
P30101	PDIA3	Protein disulfide-isomerase A3	-	+
Q15645	TRIP13	Pachytene checkpoint protein 2 homolog	-	+
P14618	PKM	Pyruvate kinase PKM	-	+
P07900-2	HSP90AA1	Isoform 2 of Heat shock protein HSP 90-alpha	-	+
P63104-1	YWHAZ	14-3-3 protein zeta/delta	-	+
Q96Q05-2	TRAPPC9	Isoform 2 of Trafficking protein particle complex subunit 9	-	+
Q9NV31	IMP3	U3 small nucleolar ribonucleoprotein protein IMP3	-	+
Q13671	RIN1	ras and Rab interactor 1	-	+
O43918-1	AIRE	autoimmune regulator	-	+
P60174	TPI1	Triosephosphate isomerase	-	+
Q14240-2	EIF4A2	Isoform 2 of Eukaryotic initiation factor 4A-II	-	+
P05089-2	ARG1	Isoform 2 of Arginase-1	-	+
Q96QA5	GSDMA	gasdermin-A	-	+
P52943-2	CRIP2	Isoform 2 of Cysteine-rich protein 2	-	+
P02545	lamin A/C	Prelamin-A/C	-	+
P02788	LTF	Lactotransferrin	-	+
P62913	RPL11	60S ribosomal protein L11	-	+
Q15828	CST6	Cystatin-M	-	+

Supplementary Table 2 Oligonucleotides used for Clonging, qRT-PCR, ChIP-PCR, EMSA and knockdown experiments

Name	Sequence
**For qRT-PCR**	
hGAPDH_FP	GGATATTGTTGCCATCAATGACC
hGAPDH_RP	AGCCTTCTCCATGGTGGTGAAGA
hHOXA9_FP	GCTTGTGGTTCTCCTCCAGTTG
hHOXA9_RP	TCCCTGGTGAGGTACATGTTGAA
hHIF-1α_FP	TCCGATGGAAGCACTAGACAAAG
hHIF-1α_RP	TGACAACTGATCGAAGGAACGTAA
hHK2_FP	CGTCTACAAGAAACACCCCCATT
hHK2_RP	ACCTCGCTCCATTTCTACCTTCA
hGLUT1_FP	GCTTCTCCAACTGGACCTCAAAT
hGLUT1_RP	TCCTCGGGTGTCTTGTCACTTT
hPDK1_FP	GGACAAAAGTGCTGAGGATGCTA
hPDK1_RP	GCGACTCATGTAGAATCGATCCA
**For clonging (pMIR-report constructs)**	
hHOXA9_3UTR_FP	CTAGTTATAAGAAAAAGGAAAAGTTGAGGGGGGGGCATTAGTGCTGATAGGAGCT
hHOXA9_3UTR_RP	CCTATCAGCACTAATGCCCCCCCCTCAACTTTTCCTTTTTCTTATAA
hHOXA9_3UTR_mut_FP	CTAGTTATAAGAAAAAGGAAAAGTTGAGGGGGGACTGCGAGTGCTGATAGGAGCT
hHOXA9_3UTR_mut_RP	CCTATCAGCACTCGCAGTCCCCCCTCAACTTTTCCTTTTTCTTATAA
**For ChIP-PCR**	
HIF-1α_HOXA9 ChIP_FP	CGTGCAGGTTTTGTTTCGTTTTA
HIF-1α_HOXA9 ChIP_RP	TGTCACGAGAACGCAGATATTATAAATG
HK2_HOXA9 ChIP_FP	GGTCAGATGCACGGTCTGTTTTA
HK2_HOXA9 ChIP_RP	CAAATCTTTTTCTTTCCCAGTTGTCT
GLUT1_HOXA9 ChIP_FP	TGGAAATTGCACCTCTCCTGATA
GLUT1_HOXA9 ChIP_RP	CAGGCTGGTCTTGAACTCTTGAC
PDK1_HOXA9 ChIP_FP	TACCCGTTACACTTTCTAAAACCAACA
PDK1_HOXA9 ChIP_RP	CCAATCTGCGTTTTCCCTGAA
HK2_HIF-1α ChIP_FP	GAGGGTTGAGGACGTGCATTTAG
HK2_HIF-1α ChIP_RP	TGAACTCCTGGGCTCAAGCA
GLUT1_HIF-1α ChIP_FP	TAGCAACAGCGAGCGTGCCG
GLUT1_HIF-1α ChIP_RP	CCCCGTCGTTTGGTCTCCT
PDK1_HIF-1α ChIP_FP	TTGCATATAGATTGAGGTCTCCTGTCT
PDK1_HIF-1α ChIP_RP	CGTGACACAGCCTCAGGAAGTC
**EMSA probes**	
HOXA9 motif_HIF-1α locus_top	CAGCAGATATTATAAAACGCGC
HOXA9 motif_HIF-1α locus_bottom	GCGCGTTTTATAATATCTGCTG
HOXA9 motif_mut_HIF-1α locus_top	CAGCAGATAGGGGAAAACGCGC
HOXA9 motif_mut_HIF-1α locus_bottom	GCGCGTTTTCCCCTATCTGCTG
HOXA9 motif_HK2 locus_top	CAGCAGGTTTTATAGGACGCGC
HOXA9 motif_HK2 locus_bottom	GCGCGTCCTATAAAACCTGCTG
HOXA9 motif_mut_HK2 locus_top	CAGCAGGTTGGGGAGGACGCGC
HOXA9 motif_mut_HK2 locus_bottom	GCGCGTCCTCCCCAACCTGCTG
HOXA9 motif_GLUT1 locus_top	CAGCAGCAAATAAATAACGCGC
HOXA9 motif_GLUT1 locus_bottom	GCGCGTTATTTATTTGCTGCTG
HOXA9 motif_mut_GLUT1 locus_top	CAGCAGCAACCCCATAACGCGC
HOXA9 motif_mut_GLUT1 locus_bottom	GCGCGTTATGGGGTTGCTGCTG
HOXA9 motif_PDK1 locus_top	CAGCAGGCAGATTGGTGACGCGC
HOXA9 motif_PDK1 locus_bottom	GCGCGTCACCAATCTGCCTGCTG
HOXA9 motif_mut_PDK1 locus_top	CAGCAGGCACCCCGGTGACGCGC
HOXA9 motif_mut_PDK1 locus_bottom	GCGCGTCACCGGGGTGCCTGCTG
**siRNAs oligos for knockdown assays**	
si-HOXA9 #1	CCUCCAGUUGAUAGAGAAA
si-HOXA9 #2	CCGGCCUUAUGGCAUUAAA
si-CRIP2 #1	UGGGCAGCUACAUCUAUGA
si-CRIP2 #2	CUGAGAAGGUGACGUCUCU
