# Supplementary material for: HOXA9 inhibits HIF-1α-mediated glycolysis through interacting with CRIP2 to repress cutaneous squamous cell carcinoma development
Source: Nat Commun. 2018 Apr 16;9:1480. doi: 10.1038/s41467-018-03914-5 (PMC5902613; doi:10.1038/s41467-018-03914-5)
Supplement: Supplementary file 3 — Description of Additional Supplementary Files [file 41467_2018_3914_MOESM3_ESM.pdf]

Description of Additional Supplementary Files

File Name: Supplementary Data 1
Description: Differentially-expressed transcripts in response to HOXA9 depletion.

File Name: Supplementary Data 2
Description: Functional annotation clustering of regulated genes in HOXA9-depleted cells.

File Name: Supplementary Data 3
Description: KEGG pathway analysis of regulated genes in HOXA9-depleted cells.
